# Green MW/US‐Assisted Construction of Pyrano[2,3‐b]pyridines and Pyrazolo[3,4‐d]pyrimidinones: Pathway Control and Antibacterial Evaluation

**DOI:** 10.1002/cbdv.71844

**Published:** 2026-10-21

**Authors:** Sultanah M. Alhunayhin, Ayman M. Algohary, Youssef O. Al‐Ghamdi, Mohamed Abdel‐Megid, Asmaa M. Fahim, Sameh A. Rizk

**Affiliations:** ^1^ Department of Chemistry, College of Science Majmaah University Al‐Zulfi, Riyadh Saudi Arabia; ^2^ Chemistry Department, College of Science Imam Mohammad Ibn Saud Islamic University (IMSIU) Riyadh Saudi Arabia; ^3^ Department of Green Chemistry National Research Centre Cairo Egypt; ^4^ Chemistry Department, Faculty of Science Ain Shams University Cairo Egypt

**Keywords:** activation‐mode selectivity, antibacterial activity, DFT analysis, fused heterocycles, green synthesis, microwave irradiation, molecular docking, pyrano[2,3‐b]pyridines, pyrazolo[3,4‐d]pyrimidinones, ultrasound‐assisted synthesis

## Abstract

A green, activation‐controlled route was examined for the preparation of biologically relevant fused N/O heterocycles, with particular emphasis on whether the mode of energy delivery can redirect the reaction pathway. A single hydrazine‐derived precursor was treated with acetic anhydride under the same nominal temperature and reaction time while applying ultrasound (US), microwave (MW), or combined MW/US activation. The three modes led selectively to different annulated products: pyrazolo[3,4‐d]pyrimidinone 5 was isolated in 88% yield under US, oxazinyl–pyrazolidinone 6 in 85% yield under MW, and the more extensively fused derivative 7 in 85% yield under combined MW/US conditions. These results indicate that pathway selection can be influenced by the physical manner in which energy is supplied without altering the reagent set. The products were assigned from FT‐IR, NMR, EI–MS, and elemental‐analysis data and were screened against representative Gram‐positive and Gram‐negative bacteria. Compound 5 gave the best overall antibacterial response, including a 32.0 ± 0.6 mm inhibition zone against *Staphylococcus aureus* and MIC values of ≤ 12.5 µg/mL. Its experimental performance was consistent with favorable docking contacts and stable molecular‐dynamics behavior, while DFT/Mulliken analysis provided a rationale for the reactive sites involved in annulation and dehydration.

## Graphical abstract

A rapid, green MW/US‐assisted route was developed for synthesizing coumarin/azacoumarin‐based fused heterocycles. It enables efficient construction of pyrano[2,3‐b]pyridines and pyrazolo[3,4‐d]pyrimidinones under mild conditions. FT‐IR, NMR, MS, and elemental analysis fully confirmed the structures of the new products. The synthesized library showed promising antibacterial activity against Gram‐positive and Gram‐negative strains. DFT descriptors and molecular docking/MD rationalized structure–activity relationships and binding interactions (e.g., PDB targets 2WJE and 3T88).

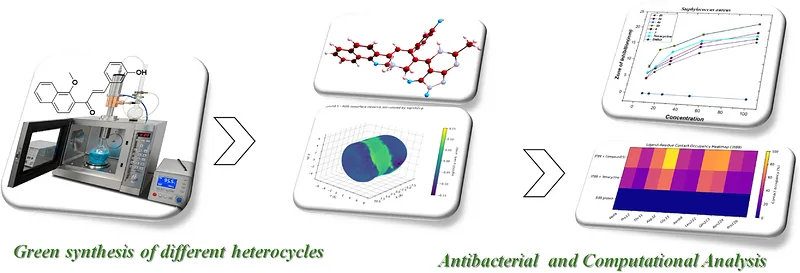

## 1 Introduction

Antimicrobial resistance continues to reduce the effectiveness of established antibiotics and has intensified the search for chemically distinct small molecules active against both Gram‐positive and Gram‐negative pathogens. Fused heterocycles containing nitrogen and oxygen are attractive in this regard because rigid conjugated cores can present several donor and acceptor sites in a defined geometry, enabling hydrogen bonding, π‐associated contacts, hydrophobic recognition, and electrostatic interactions with bacterial macromolecules [1, 2, 3, 4, 5]. Coumarin and azacoumarin frameworks are particularly useful medicinal chemistry platforms and have been associated with antimicrobial, anticancer, anti‐inflammatory, antiviral, and enzyme‐inhibitory effects. Additional ring fusion can further constrain molecular shape and create new pharmacophoric interaction points as outlined in Figure 1. Pyrano[2,3‐b]pyridines combine a pyridine ring with an oxygen‐containing fused system and can accommodate carbonyl, amino, cyano, ester, phenolic, and aromatic functionalities within one scaffold. Pyrazole‐containing systems are likewise prominent in bioactive‐molecule design; notably, pyrazolo[3,4‐d]pyrimidinones provide a nitrogen‐rich surface capable of strong hydrogen‐bonding and electronic interactions. Consequently, hybrid architectures that incorporate pyridone, pyran, pyrazole, pyrimidinone, and oxazine elements may offer complementary interaction motifs and useful antibacterial profiles [1, 6, 7, 8, 9, 10, 11].

**FIGURE 1:**
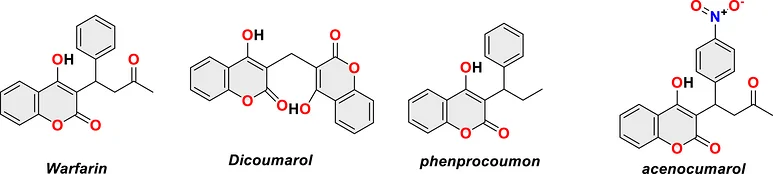
Representative drugs containing a coumarin moiety.

The development of energy‐efficient reaction methods has also become an important objective in heterocyclic synthesis [12, 13, 14, 15, 16, 17, 18]. Microwave activation can provide rapid volumetric dielectric heating and may preferentially excite polar or ionic species, whereas ultrasound improves interfacial contact and micromixing through acoustic cavitation and the transient formation of highly energetic local environments [19, 20, 21, 22, 23, 24, 25, 26]. Applying the two modes together can therefore influence cyclization, annulation, and dehydration while maintaining relatively mild bulk conditions. Most studies using MW or US, however, emphasize shorter reaction times or improved isolated yields. Much less attention has been given to the possibility that the activation mode itself may change product selectivity when the precursor and reagent composition are kept constant [2, 27, 28, 29].

Accordingly, the present study was designed to establish an activation mode dependent synthetic platform for fused pyrano[2,3‐b]pyridine and pyrazolo[3,4‐d]pyrimidinone derivatives. The central comparison involved US, MW, and combined MW/US treatment at matched reaction times and temperatures so that differences in product formation could be related primarily to the type of physical activation. The experimentally observed divergence was interpreted mechanistically with support from DFT/Mulliken charge calculations. The synthesized compounds were also examined for activity against representative Gram‐positive and Gram‐negative bacteria, and docking together with molecular‐dynamics calculations was used to assess plausible ligand–target interactions and the stability of the corresponding complexes. This combined approach connects pathway control under greener activation conditions with mechanistic analysis and antibacterial screening.

## 2 Experimental Section

### 2.1 Instruments

Melting ranges were measured in open capillary tubes and are reported without correction. FT‐IR measurements were obtained as KBr pellets on a Shimadzu FT‐IR 8400S instrument, with band positions expressed in cm^−^^1^. NMR spectra were acquired in DMSO‐d_6_ using TMS as the reference; the operating frequencies were 300 MHz for ^1^H and 125 MHz for ^1^ ^3^C. Electron‐impact mass spectra were collected at 70 eV on a Shimadzu GCMS‐QP‐1000 EX system, and elemental compositions were determined with an automated CHN analyzer. Sonochemical experiments employed a Toshcon SW‐4 unit (30 kHz, 500 W). Microwave‐only and combined MW/US reactions were performed in an XH‐300UL reactor (Xianzhi Technology, Beijing, China; maximum MW power 950 W). The stated reaction temperature was monitored during each experiment, and conversion was followed by TLC with an appropriate eluent. Unless a specific purification step is noted, commercial reagents and solvents were used directly.

### 2.2 Chemicals and Reagents

Reagents and solvents were sourced from Aldrich Fine Chemical Company and used without additional purification. The principal starting materials and auxiliaries were KOH, *m*‐hydroxybenzaldehyde, 2‐acetyl‐1‐methoxynaphthalene, ethyl acetoacetate, ethyl cyanoacetate, diethyl malonate, ammonium acetate, hydrazine hydrate, and acetic anhydride; ethanol served mainly as the reaction and recrystallization medium. Table S1 compiles the appearance, isolated yield, melting range, FT‐IR bands, ^1^H/^1^ ^3^C NMR shifts, EI–MS ions, and elemental analysis data for the synthesized products. Fragmentation information is collected in Table S2, while the corresponding spectra are provided in Figures S1–S19.

### 2.3 Synthesis of (E)‐3‐(3‐Hydroxyphenyl)‐1‐(1‐methoxynaphthalen‐2‐yl)prop‐2‐en‐1‐one (1)

2‐Acetyl‐1‐methoxynaphthalene (2.0 g), 3‐hydroxybenzaldehyde (1.5 g), and KOH (2.0 g) were mixed with a few drops of water and subjected to grinding under ultrasound for 20 min. The mixture gradually became light yellow [30]. TLC was used to confirm completion, after which water was added, and the precipitated material was collected by filtration, washed with water, dried, and recrystallized from ethanol. (E)‐3‐(3‐Hydroxyphenyl)‐1‐(1‐methoxynaphthalen‐2‐yl)prop‐2‐en‐1‐one (1) was isolated as pale‐yellow crystals (99% yield; mp 88°C–90°C). Electron‐impact MS (70 eV) gave m/z 304 [M]^+^. Elemental analysis (calculated/found) for C_20_H_16_O_3_ (304.34): C, 78.93; H, 5.30%. Found: C, 78.96; H, 5.32%. The ^1^ ^3^C NMR shifts (125 MHz, DMSO‐d_6_), listed from downfield to upfield, were *δ* 188.9, 150.5, 146.4, 145.3, 140.9, 136.6, 130.1, 129.4, 128.1, 121.5, 114.3, 55.0. The ^1^H NMR signals (300 MHz, DMSO‐d_6_), reported from downfield to upfield, were δ 9.22 (s, 1H, OH, D_2_O‐exchangeable), 8.02 (d, 1H, CH═, *J* = 16.2 Hz), 7.82 (d, 1H, CH═, *J* = 16.2 Hz), 6.80–8.02 (m, Ar─H and olefinic H), 3.59 (s, 3H, OCH_3_). FT‐IR (KBr) bands, regrouped by assignment, were ν_max_ (cm^−^^1^): 1600 (C═C); 1679 (C═O).

### 2.4 General Procedure for the Synthesis of Nicotinate/Pyridone Derivatives 2a–2c

For compounds 2a–2c, chalcone 1 (1 mmol) was combined with 1 mmol of the required active‐methylene reagent (ethyl cyanoacetate, ethyl acetoacetate, or diethyl malonate) in the presence of ammonium acetate. The mixture was sonicated for 25–30 min and monitored by TLC. Once the starting material had been consumed, the reaction was allowed to cool and the precipitated product was isolated, washed, dried, and recrystallized from ethanol [30].

Ethyl 2‐amino‐4‐(3‐hydroxyphenyl)‐6‐(1‐methoxynaphthalen‐2‐yl)nicotinate (2a) was isolated as yellow crystals (85% yield; m.p 176°C–178°C). Electron‐impact MS (70 eV) gave m/z 414 [M]^+^, 388 [M − 26]^+^. Elemental analysis (calculated/found) for C_25_H_22_N_2_O_4_ (414.46): C, 72.45; H, 5.35; N, 6.76%. Found: C, 72.49; H, 5.37; N, 6.70%. The ^1^ ^3^C NMR shifts (125 MHz, DMSO‐d_6_), listed from downfield to upfield, were *δ* 167.0, 150.5, 146.4, 145.3, 139.9, 136.6, 131.0, 128.5, 127.0, 121.0, 55.0, 29.0. The ^1^H NMR signals (300 MHz, DMSO‐d_6_), reported from downfield to upfield, were δ 9.41 (s, 1H, OH, D_2_O‐exchangeable), 8.71 (s, 2H, NH_2_, D_2_O‐exchangeable), 7.63–8.54 (m, 12H, Ar─H), 4.14 (q, 2H, CH_2_), 1.21 (t, 3H, CH_3_). FT‐IR (KBr) bands, regrouped by assignment, were ν_max_ (cm^−^^1^): 1732 and 1669 (C═O); 3045 (Ar─H); 3479 (OH).

Ethyl 4‐(3‐hydroxyphenyl)‐2‐methyl‐6‐(1‐methoxynaphthalen‐2‐yl)nicotinate (2b) was isolated as light‐yellow crystals (84% yield; m.p 184°C–186°C). Electron‐impact MS (70 eV) gave m/z 413 [M]^+^. Elemental analysis (calculated/found) for C_26_H_23_NO_4_ (413.47): C, 75.53; H, 5.61; N, 3.39%. Found: C, 75.55; H, 5.58; N, 3.42%. The ^1^H NMR signals (300 MHz, DMSO‐d_6_), reported from downfield to upfield, were *δ* 8.95–8.97 (br s, 1H, OH, D_2_O‐exchangeable), 7.47–8.19 (m, 12H, Ar─H), 4.02 (q, 2H, CH_2_), 3.67 (s, 3H, OCH_3_), 2.28 (s, 3H, CH_3_), 1.03 (t, 3H, CH_3_). FT‐IR (KBr) bands, regrouped by assignment, were ν_max_ (cm^−^^1^): 1733, 1725, and 1674 (C═O); 3045 (Ar─H); 3316 (OH).

Ethyl 4‐(3‐hydroxyphenyl)‐6‐(1‐methoxynaphthalen‐2‐yl)‐2‐oxo‐1,2‐dihydropyridine‐3‐carboxylate (2c) was isolated as light‐yellow crystals (71% yield; m.p 176°C–178°C). Electron‐impact MS (70 eV) gave m/z 415 [M]^+^. Elemental analysis (calculated/found) for C_25_H_21_NO_5_ (415.45): C, 72.28; H, 5.10; N, 3.37%. Found: C, 72.32; H, 5.12; N, 3.35%. The ^1^ ^3^C NMR shifts (125 MHz, DMSO‐d_6_), listed from downfield to upfield, were *δ* 166.7, 147.5, 142.4, 138.6, 135.8, 132.3, 129.5, 128.2, 127.3, 57.4, 31.1. The ^1^H NMR signals (300 MHz, DMSO‐d_6_), reported from downfield to upfield, were δ 10.02 (s, 1H, OH, D_2_O‐exchangeable), 7.63–8.54 (m, 12H, Ar─H), 3.99 (q, 2H, CH_2_), 3.58 (s, 3H, OCH_3_), 0.95 (t, 3H, CH_3_). FT‐IR (KBr) bands, regrouped by assignment, were ν_max_ (cm^−^^1^): 1733 (C═O); 3045 (Ar─H); 3411 (OH).

### 2.5 General Procedure for the Synthesis of Pyrano[2,3‐b]Pyridine Derivatives 3a–3c

Each pyrano[2,3‐b]pyridine derivative 3a–3c was prepared from the corresponding nicotinate/pyridone precursor 2a–2c (1 mmol), the appropriate active‐methylene component (1 mmol), and ammonium acetate. After thorough mixing, ethanol (10 mL) was added and the suspension was sonicated at 30°C until TLC showed disappearance of the precursor. The solid formed under these conditions was collected, washed, dried, and purified by recrystallization from ethanol.

4‐Amino‐5‐(3‐hydroxyphenyl)‐7‐(1‐methoxynaphthalen‐2‐yl)‐2‐oxo‐2H‐pyrano[2,3‐b]pyridine‐3‐carbonitrile (3a) was isolated as light‐yellow crystals (82% yield; m.p 176°C–178°C). Electron‐impact MS (70 eV) gave m/z 435 [M]^+^. Elemental analysis (calculated/found) for C_26_H_17_N_3_O_4_ (435.44): C, 71.72; H, 3.94; N, 9.65%. Found: C, 71.67; H, 3.73; N, 9.36%. The ^1^ ^3^C NMR shifts (125 MHz, DMSO‐d_6_), listed from downfield to upfield, were *δ* 168.7, 151.5, 147.4, 133.6, 131.8, 130.3, 128.5, 127.2, 126.3, 113.5, 61.4. The ^1^H NMR signals (300 MHz, DMSO‐d_6_), reported from downfield to upfield, were δ 9.26 (s, 1H, OH, D_2_O‐exchangeable), 8.14 (s, 2H, NH_2_, D_2_O‐exchangeable), 7.48–7.86 (m, 11H, Ar─H). FT‐IR (KBr) bands, regrouped by assignment, were ν_max_ (cm^−^^1^): 1734 (C═O); 2270 (C≡N); 3055 (Ar─H); 3404 and 3324 (NH_2_).

3‐Acetyl‐5‐(3‐hydroxyphenyl)‐4‐methyl‐7‐(1‐methoxynaphthalen‐2‐yl)‐2H‐pyrano[2,3‐b]pyridin‐2‐one (3b) was isolated as yellow crystals (90% yield; m.p 184°C–186°C). Electron‐impact MS (70 eV) gave m/z 451 [M]^+^. Elemental analysis (calculated/found) for C_28_H_21_NO_5_ (451.48): C, 74.49; H, 4.69; N, 3.10%. Found: C, 74.55; H, 4.54; N, 3.32%. The ^1^ ^3^C NMR shifts (125 MHz, DMSO‐d_6_), listed from downfield to upfield, were *δ* 202.3, 165.7, 151.5, 140.4, 135.6, 132.8, 130.3, 127.5, 125.2, 123.3, 58.4, 42.1. The ^1^H NMR signals (300 MHz, DMSO‐d_6_), reported from downfield to upfield, were δ 9.32 (s, 1H, OH, D_2_O‐exchangeable), 7.47–8.19 (m, 11H, Ar─H), 4.01 (s, 3H, OCH_3_), 2.53 (s, 3H, COCH_3_), 2.21 (s, 3H, CH_3_). FT‐IR (KBr) bands, regrouped by assignment, were ν_max_ (cm^−^^1^): 1720 (C═O); 3045 (Ar─H); 3211 (OH).

Ethyl 5‐(3‐hydroxyphenyl)‐7‐(1‐methoxynaphthalen‐2‐yl)‐2,4‐dioxo‐2H‐pyrano[2,3‐b]pyridine‐3‐carboxylate (3c) was isolated as light‐yellow crystals (84% yield; mp 184°C–186°C). Electron‐impact MS (70 eV) gave m/z 483 [M]^+^, 453 [M − 30]^+^. Elemental analysis (calculated/found) for C_28_H_21_NO_7_ (483.48): C, 69.56; H, 4.38; N, 2.90%. Found: C, 69.57; H, 4.35; N, 2.88%. The ^1^H NMR signals (300 MHz, DMSO‐d_6_), reported from downfield to upfield, were δ 9.25 (s, 1H, OH, D_2_O‐exchangeable), 7.47–8.19 (m, 11H, Ar─H), 5.92 (s, 1H, CH), 3.99 (q, 2H, CH_2_), 3.58 (s, 3H, OCH_3_), 0.95 (t, 3H, CH_3_). FT‐IR (KBr) bands, regrouped by assignment, were ν_max_ (cm^−^^1^): 1736 and 1676 (C═O); 3045 (Ar─H); 3428 (OH).

### 2.6 General Procedure for the Reaction of Azacoumarin Derivatives With Hydrazine Hydrate

The selected azacoumarin precursor 3a or 3b (1 mmol) and hydrazine hydrate (1 mmol) were dissolved in ethanol (10 mL) and exposed to simultaneous MW/US activation at 50°C for 5 min. TLC was used to follow conversion. The completed reaction mixture was discharged onto crushed ice, after which the precipitate was filtered, washed, dried, and recrystallized from ethanol to furnish 4a or 4b.

(Z)‐3‐[Amino(3‐amino‐5‐oxo‐1,5‐dihydro‐4H‐pyrazol‐4‐ylidene)methyl]‐4‐(3‐hydroxyphenyl)‐6‐(1‐methoxynaphthalen‐2‐yl)pyridin‐2(1H)‐one (4a) was isolated as light‐yellow crystals (77% yield; mp 176°C–178°C). Electron‐impact MS (70 eV) gave m/z 467 [M]^+^, 388 [M − 79]^+^. Elemental analysis (calculated/found) for C_26_H_21_N_5_O_4_ (467.49): C, 66.80; H, 4.53; N, 14.98%. Found: C, 66.74; H, 4.43; N, 14.76%. The ^1^ ^3^C NMR shifts (125 MHz, DMSO‐d_6_), listed from downfield to upfield, were *δ* 166.7, 164.2, 149.5, 146.4, 139.6, 133.8, 131.3, 127.5, 126.2, 122.3, 59.4. The ^1^H NMR signals (300 MHz, DMSO‐d_6_), reported from downfield to upfield, were δ 10.86 (s, 2H, NH, D_2_O‐exchangeable), 9.41 (s, 1H, OH, D_2_O‐exchangeable), 8.14 (s, 2H, NH_2_, D_2_O‐exchangeable), 7.48–7.86 (m, 11H, Ar─H), 3.38 (s, 3H, OCH_3_). FT‐IR (KBr) bands, regrouped by assignment, were ν_max_ (cm^−^^1^): 1613 (C═N); 1672–1663 (C═O); 3055 (Ar─H); 3294 and 3183 (NH/NH_2_).

4‐(3‐Hydroxyphenyl)‐3‐[1‐(1,5‐dihydro‐3‐methyl‐5‐oxo‐4‐pyrazolylidene)ethyl]‐6‐(1‐methoxynaphthalen‐2‐yl)pyridin‐2(1H)‐one (4b) was isolated as light‐yellow crystals (82% yield; m.p 184°C–186°C). Electron‐impact MS (70 eV) gave m/z 465 [M]^+^. Elemental analysis (calculated/found) for C_28_H_23_N_3_O_4_ (465.51): C, 72.25; H, 4.98; N, 9.03%. Found: C, 72.23; H, 4.96; N, 9.00%. The ^1^H NMR signals (300 MHz, DMSO‐d_6_), reported from downfield to upfield, were δ 11.21 (s, 1H, NH, D_2_O‐exchangeable), 9.41 (s, 1H, OH, D_2_O‐exchangeable), 7.46–8.18 (m, 11H, Ar─H), 3.35 (s, 3H, OCH_3_), 2.51 (s, 3H, CH_3_). FT‐IR (KBr) bands, regrouped by assignment, were νmax (cm^−^^1^): 1674 (C═O); 3045 (Ar─H).

### 2.7 Divergent Synthesis of Annulated Derivatives 5–7 From Compound 4a

Compound 4a (1 mmol) was mixed thoroughly with acetic anhydride (0.02 mol) and then transferred to a round‐bottom flask containing ethanol (10 mL). Three activation protocols were compared independently at the same bulk conditions (20 min, 30°C): ultrasound, microwave, and sequential/combined US–MW treatment. Reaction progress was checked by TLC. Each reaction was finally poured onto crushed ice, and the isolated solid was filtered, washed, dried, and recrystallized from ethanol, providing the corresponding annulated product 5, 6, or 7 [30].

4‐[4‐(3‐Hydroxyphenyl)‐6‐(1‐methoxynaphthalen‐2‐yl)‐2‐oxo‐1,2‐dihydropyridin‐3‐yl]‐6‐methyl‐2,5‐dihydro‐3H‐pyrazolo[3,4‐d]pyrimidin‐3‐one (5) was isolated as light‐yellow crystals (88% yield; mp 176°C–178°C). Electron‐impact MS (70 eV) gave m/z 491 [M]^+^, 424 [M − 67]^+^. Elemental analysis (calculated/found) for C_28_H_21_N_5_O_4_ (491.51): C, 68.42; H, 4.31; N, 14.25%. Found: C, 68.40; H, 4.32; N, 14.23%. The ^1^ ^3^C NMR shifts (125 MHz, DMSO‐d_6_), listed from downfield to upfield, were *δ* 166.88, 156.69, 145.5, 143.4, 131.6, 129.13, 129.01, 125.39, 119.54, 116.45, 116.23, 58.16, 43.58, 21.78, 20.63. The ^1^H NMR signals (300 MHz, DMSO‐d_6_), reported from downfield to upfield, were δ 11.92–12.21 (s, 1H, NH, D_2_O‐exchangeable), 10.85 (s, 1H, NH, D_2_O‐exchangeable), 9.22 and 10.32 (s, 2H, OH and NH, D_2_O‐exchangeable), 7.16–8.88 (m, Ar─H), 4.91 (s, 1H, pyridine‐H), 3.28 (s, 3H, OCH_3_), 1.48 (s, 3H, CH_3_). FT‐IR (KBr) bands, regrouped by assignment, were ν_max_ (cm^−^^1^): 1612 (C═N); 1686–1663 (C = O); 3057 (Ar─H); 3310 (NH); 3404 (OH).

4‐[5‐(3‐Hydroxyphenyl)‐2‐methyl‐7‐(1‐methoxynaphthalen‐2‐yl)‐4H‐pyrido[3,2‐e][1,3]oxazin‐4‐ylidene]‐5‐iminopyrazolidin‐3‐one (6) was isolated as light‐yellow crystals (85% yield; mp 176°C–178°C). Electron‐impact MS (70 eV) gave m/z 491 [M]^+^, 428 [M − 63]^+^. Elemental analysis (calculated/found) for C_28_H_21_N_5_O_4_ (491.51): C, 68.42; H, 4.31; N, 14.25%. Found: C, 68.40; H, 4.28; N, 14.23%. The ^1^ ^3^C NMR shifts (125 MHz, DMSO‐d_6_), listed from downfield to upfield, were *δ* 174.11, 161.68, 148.35, 140.84, 133.79, 130.29, 129.79, 129.05, 126.61, 125.35, 123.25, 118.86, 118.06, 117.67, 58.86, 36.44, 20.63. The ^1^H NMR signals (300 MHz, DMSO‐d_6_), reported from downfield to upfield, were δ 11.47 (br s, 2H, imine‐NH and NHCO, D_2_O‐exchangeable), 7.21–8.02 (m, Ar─H), 6.82 (s, 1H, NH, D_2_O‐exchangeable), 6.19 (s, 1H, pyridine‐H), 3.18 (s, 3H, OCH_3_), 2.07 (s, 3H, CH_3_). FT‐IR (KBr) bands, regrouped by assignment, were ν_max_ (cm^−^^1^): 1630–1586 (C═N); 1673 and 1654 (C═O); 3054 and 3020 (Ar─H); 3330, 3164, and 3113 (NH); 3434 (OH) [30].

1‐(3‐Hydroxyphenyl)‐6‐methyl‐3‐(1‐methoxynaphthalen‐2‐yl)‐9,10‐dihydro‐6H,11H‐pyrazolo[3′,4′:3,4]pyrazolo[1,5‐c]pyrido[3,2‐e][1,3]oxazin‐11‐one (7) was isolated as yellow crystals (85% yield; m.p 176°C–178°C). Electron‐impact MS (70 eV) gave m/z 491 [M]^+^, 487 [M − 4]^+^. Elemental analysis (calculated/found) for C_28_H_21_N_5_O_4_ (491.51): C, 68.42; H, 4.31; N, 14.25%. Found: C, 68.40; H, 4.29; N, 14.23%. The ^1^ ^3^C NMR shifts (125 MHz, DMSO‐d_6_), listed from downfield to upfield, were *δ* 191.62, 162.26, 157.25, 140.42, 137.82, 136.22, 135.92, 131.86, 129.21, 128.36, 127.76, 126.50, 126.11, 121.36, 120.19, 118.0, 113.03, 69.75, 20.73, 10.44. The ^1^H NMR signals (300 MHz, DMSO‐d_6_), reported from downfield to upfield, were δ 10.65 (s, 1H, NH, D_2_O‐exchangeable), 9.49 and 9.57 (s, 2H, OH and NH, D_2_O‐exchangeable), 6.79–7.90 (m, Ar─H and pyridine‐H), 4.51 (s, 1H, oxazine‐H), 3.37 (s, 3H, OCH_3_), 2.40 (s, 3H, CH_3_). FT‐IR (KBr) bands, regrouped by assignment, were ν_max_ (cm^−^^1^): 1631–1619 (C═N); 1657 (C═O); 3055 (Ar─H); 3468 and 3345 (NH/OH).

### 2.8 Biological Activities

#### 2.8.1 Antibacterial Activity

Antibacterial testing was carried out for compounds 2b, 3a, 4a, 5, 6, and 7 using two Gram‐positive strains, *Staphylococcus aureus* ATCC 6538 and *Enterococcus faecalis* ATCC 29212, and two Gram‐negative strains, *Pseudomonas aeruginosa* ATCC 9027 and *Escherichia coli* ATCC 10536. After full identification at first mention, the abbreviated species names were used throughout. Tetracycline served as the reference antibacterial agent, whereas DMSO was included as the solvent control.

Agar‐well diffusion was used as an initial screening assay. Overnight cultures were adjusted to approximately 0.5 McFarland and spread uniformly over sterile Mueller–Hinton agar. Circular wells (6 mm) were made with a sterile borer and loaded with 100 µL of test solution at 100, 50, or 25 µg/mL. Following incubation at 37°C for 24 h, inhibition diameters were recorded in millimeters; measurements were performed in triplicate and are reported as mean ± SD. Quantitative MIC values were then obtained by broth microdilution in 96‐well plates in accordance with CLSI/EUCAST guidance. Twofold serial dilutions were prepared in Mueller–Hinton broth, and the inoculum was adjusted to approximately 5 × 10^5^ CFU/mL per well after standardization to 0.5 McFarland. Growth, sterility, solvent, and tetracycline controls were included. Plates were incubated at 37°C for 18–24 h, and the MIC was taken as the lowest concentration with no visible bacterial growth. MIC experiments were likewise conducted in triplicate.

Because zone size in an agar assay depends partly on diffusion through the solid medium, the diffusion experiment was treated as a qualitative or semi‐quantitative screen. Broth‐microdilution MIC values were therefore used as the principal numerical measure of antibacterial potency [31].

#### 2.8.2 Molecular Docking Analysis

Docking calculations were performed with AutoDock Vina 1.2.3 through MOE 2019.01, and the resulting binding poses were inspected in Discovery Studio Visualizer 4.5 [32, 33]. Two bacterial targets were considered: *Escherichia coli* MenB bound to the substrate analogue OSB‐NCoA (PDB 3T88) [34] and the Cps4B tyrosine phosphatase from *Streptococcus pneumoniae* TIGR4 (PDB 2WJE) [35]. Structures downloaded from the Protein Data Bank were prepared by deleting crystallographic water and the co‐crystallized ligand, adding polar hydrogens and Gasteiger charges, and applying AMBER99‐based optimization. Ligands were built in ChemDraw 20.0 and minimized with MMFF94. The search region was centered on the native‐ligand pocket using a 40 × 40 × 40 Å box, 1.0 Å spacing, and exhaustiveness 8 [21, 36]. Ten runs were evaluated per ligand; ranking considered the Vina binding score together with hydrogen‐bonding, hydrophobic, and electrostatic contacts. Method validation was based on re‐docking of the crystallographic ligand, for which an RMSD not exceeding 2.0 Å was required [22].

#### 2.8.3 Molecular Dynamics Analysis

The highest‐ranked protein–ligand complexes were advanced to 100 ns all‐atom MD simulations in GROMACS 2022.2. AMBER14SB parameters were used for the proteins, GAFF2 for the ligands, and ligand charges were generated with the Antechamber/AM1‐BCC procedure [37]. Each complex was placed in a triclinic box of explicit TIP3P water and neutralized with Na^+^/Cl^−^ to give 0.15 M ionic strength. Steepest‐descent minimization was allowed to proceed for up to 50,000 steps using a convergence threshold of 10^−^^4^ kJ mol^−^^1^ nm^−^^1^. Equilibration comprised 100 ps in the NVT ensemble and a further 100 ps under NPT conditions at 1 bar and 300 K, using the Berendsen thermostat and Parrinello–Rahman barostat. Covalent bonds were constrained with LINCS, long‐range electrostatics were treated by PME with a 1.0 nm cutoff, and the production trajectory used a 2 fs integration step for 100 ns with coordinates stored every 10 ps [38].

Trajectory analysis focused on both structural persistence and binding energetics. Probability distributions were generated for RMSD, radius of gyration (R_g_), and selected hydrogen‐bond distances to assess the degree of conformational confinement. Dominant collective motions were examined by PCA of the Cα covariance matrix and were projected onto free‐energy landscapes using −kBT ln P. Binding free energies and residue‐wise contributions were estimated by MM/GBSA using the AMBER14SB/TIP3P and GAFF2 description, igb = 5, and 0.15 M NaCl; equilibrated structures were sampled at one frame every 100 ps.

### 2.9 Theoretical Investigation

Quantum‐chemical calculations were carried out in Gaussian 09 W using B3LYP/6‐311G(d), with geometry optimization by the Berny algorithm and without imposed symmetry [39, 40]. Conceptual‐DFT descriptors were obtained from Equations (1)–(9) [41, 42, 43, 44]. Site‐specific reactivity was considered through condensed finite‐difference Fukui functions: ***f^+^k = qk(N + 1) − qk(N)*** for susceptibility toward nucleophilic attack and ***f^−^k = qk(N) − qk(N − 1)*** for susceptibility toward electrophilic attack. In these expressions, qk denotes the atomic charge of atom k in the neutral, anionic, or cationic state [45]. The resulting indices were used to compare the relative preference of individual atomic centers for reaction.

(1) $$\Delta E=E_{LUMO}-E_{HOMO}$$

(2) $$x=\frac{-\left(E_{HOMO}+E_{LUMO}\right)}{2}$$

(3) $$\eta =\frac{\left(E_{LUMO}+E_{HOMO}\right)}{2}$$

(4) σ=1/η

(5) Pi=−X

(6) S=1/2η

(7) $$\omega =Pi^{2}/2$$

(8) ΔNmax=−Pi/η

(9) $$f_{k}^{0}=\frac{q_{k}\left(N+1\right)-q_{k}\left(N+1\right)}{2}$$

## 3 Results and Discussion

### 3.1 Chemistry

Chalcone 1, (E)‐3‐(3‐hydroxyphenyl)‐1‐(1‐methoxynaphthalen‐2‐yl)prop‐2‐en‐1‐one, is generated by the expected base‐promoted Claisen–Schmidt sequence. Deprotonation of the acetyl‐naphthalene component by KOH produces an enolate that adds to m‐hydroxybenzaldehyde; dehydration of the resulting β‐hydroxy ketone then establishes the conjugated α,β‐unsaturated carbonyl system shown in Scheme 1. At the B3LYP level, E_HOMO_ and E_LUMO_ were −0.19861 and −0.08247 a.u. (−5.40 and −2.24 eV), respectively, corresponding to Δ*E*_gap_ = 0.11614 a.u. (≈3.16 eV). The derived descriptors were *μ* = −3.82 eV, *χ* = 3.82 eV, *η* = 1.58 eV, and *ω* = 4.63 eV; the converged SCF energy was −996.956372 a.u. and the dipole moment was 2.268 D. Mulliken populations located the largest negative charges on O23 (−0.611957), O15 (−0.514959), and O4 (−0.366740), identifying these atoms as the most electron‐rich donor/acceptor sites. The most positive carbons were C16 (+0.366649), C5 (+0.250997), and C19 (+0.236821), consistent with enhanced electrophilic character at these positions. Numerical Fukui values would require separate N ± 1 single‐point calculations; therefore, the neutral‐state charge pattern is used here only as a qualitative guide to the most probable reactive centers.

**SCHEME 1:**
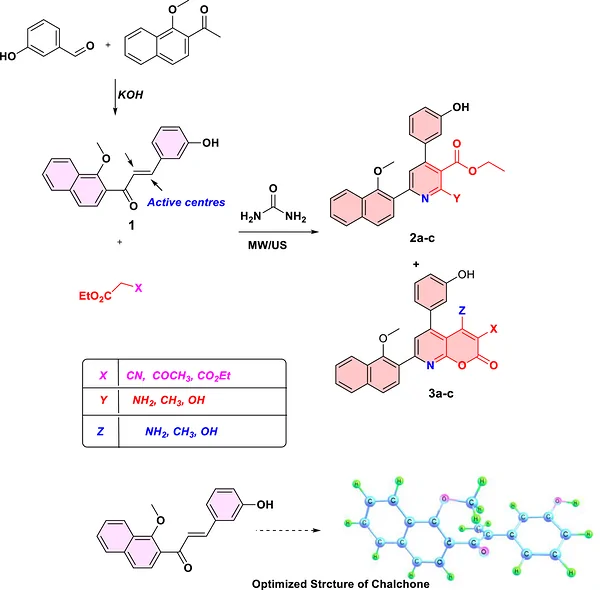
Synthetic route to chalcone 1 through KOH‐catalyzed Claisen–Schmidt condensation, followed by MW/US‐assisted multicomponent cyclocondensation of 1 with urea and active methylene esters.

In the multicomponent step, chalcone 1 functions as a Michael acceptor toward the enolate generated from ethyl cyanoacetate, ethyl acetoacetate, or diethyl malonate. Addition at the β‐carbon first establishes a new C─C bond and gives an open‐chain intermediate enriched in carbonyl and nucleophilic sites. Condensation involving urea, followed by intramolecular attack, proton transfer, water loss, and tautomerization, then furnishes the nicotinate/pyridone products 2a–2c and ultimately the pyrano[2,3‐b]pyridines 3a–3c represented in Scheme 1. Conversion of 2a–2c to 3a–3c under ultrasound in the presence of ammonium acetate is consistent with a related addition–lactonization sequence: reaction of the active‐methylene partner at the electrophilic site of 2 generates a multifunctional intermediate, and subsequent ring closure through the oxygen center creates the fused six‐membered pyran ring. The substituent delivered by the active‐methylene reagent is retained as the cyano, acetyl, or ester functionality shown in Scheme 2. For 3a, the assignment is supported by NH_2_ bands at 3272 and 3311 cm^−^^1^ together with absorptions at 2223 and 1732 cm^−^^1^ for C≡N and the lactone C═O group, respectively.

**SCHEME 2:**
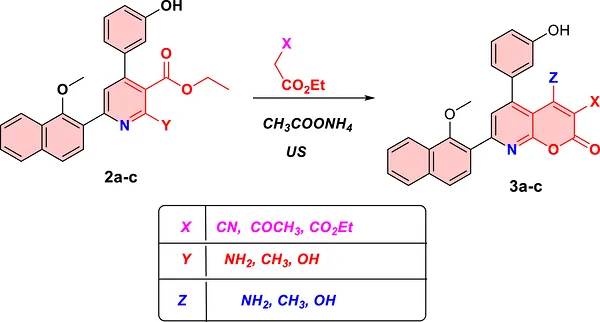
Ultrasound‐assisted conversion of 2a–2c into fused pyrano[2,3‐b]pyridine derivatives 3a–3c through reaction with active methylene esters in the presence of CH_3_COONH_4_.

Further support for 3a was obtained from its ^1^H NMR spectrum: the pyran proton appeared at δ 5.92 ppm and the exchangeable NH_2_ group as a broad signal at δ 8.14 ppm. The absence of the pyridine NH resonance expected near δ 9.8 ppm is also compatible with formation of the fused pyrano[2,3‐b]pyridine system. In the following transformation, hydrazine hydrate reacts with 3a–3c under combined MW/US conditions. Nucleophilic attack at the activated pyranone/lactone carbonyl is expected to open the oxygen‐containing ring and generate an acyl‐hydrazide‐type intermediate, setting the stage for formation of the nitrogen‐rich products 4a and 4b.

Cyclization is then promoted by the terminal hydrazide –NH_2_ group, which can condense intramolecularly with a neighboring electrophilic carbonyl or conjugated C═C site. Ring closure accompanied by proton redistribution, dehydration, and tautomerization produces the fused pyrazolone–pyridone core. The electron‐withdrawing group already present in the pyrano[2,3‐b]pyridine precursor remains embedded in the product, while the Z substituent is carried through as NH_2_ or CH_3_ according to the starting member of the series (Scheme 3).

**SCHEME 3:**
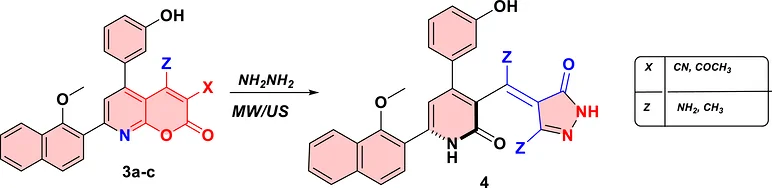
MW/US‐assisted transformation of pyrano[2,3‐b]pyridine derivatives 3a–3c into the fused hydrazine‐derived heterocycle 4 through reaction with hydrazine hydrate.

Several nucleophilic and electrophilic centers coexist in 4a, notably the pyridone/amide carbonyl group, the exocyclic amino functionality, and the adjacent N─N‐containing fragment. Acetic anhydride is therefore expected to generate N‐acetylated species and, through lactam–lactim equilibration, possibly an O‐acetylated form. Such activation places an acyl center in a favorable geometry for intramolecular attack and ring closure. Under sonication, efficient mixing and cavitation‐assisted local activation favor cyclocondensation, proton transfer, and dehydration to give pyrazolo[3,4‐d]pyrimidinone 5 (Scheme 4). Spectroscopically, 5 showed broad OH/NH absorption in the 3347–3179 cm^−^^1^ range and carbonyl bands at 1668–1652 cm^−^^1^; broadening is consistent with hydrogen bonding in the fused system. Its ^1^H NMR spectrum contained exchangeable resonances at δ 9.22 and 10.32 ppm (OH/pyrazole NH), 10.85 ppm (pyridone NH), and 11.92–12.21 ppm (pyrimidinone NH), supporting the assigned fused framework.

**SCHEME 4:**
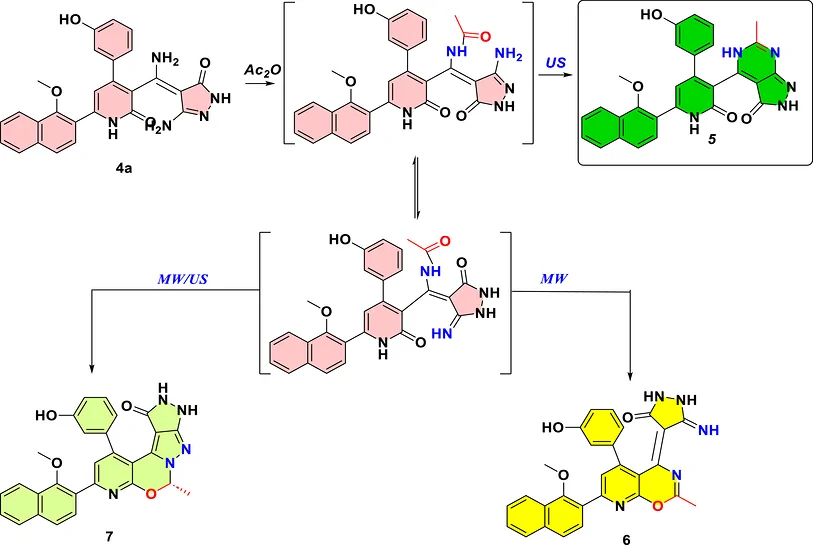
Acetic‐anhydride‐mediated acetylation/cyclodehydration of 4a to generate fused heterocycle 5 through an acylated intermediate, with condition‐controlled divergence to products 6 under MW irradiation and 7 under combined MW/US irradiation.

The product selectivity is best interpreted by separating the measured bulk temperature from the local physical consequences of the different activation modes. Ultrasound transfers energy through acoustic cavitation, creating short‐lived regions of intense shear, local heating, and rapid micromixing. These effects can accelerate contact between 4a and acetic anhydride and favor fast intramolecular cyclization/dehydration to 5. Microwave irradiation, in contrast, couples efficiently with polar bonds and ionic or highly polarized intermediates through dielectric heating; this can change the relative rates at which acetylated or enolized forms are generated and consumed and thereby favor the pathway leading to 6. When MW and US are applied together, dielectric activation is accompanied by cavitation‐enhanced mass transport, potentially lowering barriers for additional annulation, proton transfer, dehydration, and aromatization and thus favoring 7. Because the time and nominal temperature were held constant, the different product distributions are more reasonably assigned to activation‐mode‐dependent kinetics than to a simple change in bulk temperature. The proposed interpretation remains mechanistic rather than directly observed, but it is consistent with the product distribution, spectroscopic assignments, and DFT/Mulliken analysis.

For the US pathway, cavitation and rapid local mixing are proposed to drive cyclization of the acetyl‐activated intermediate from 4a. Intramolecular attack by a neighboring ring nitrogen, followed by acetic‐acid loss and proton/π‐bond reorganization, gives the conjugated N‐fused product 5, in which the acetyl‐derived carbon becomes part of a methyl‐substituted C═N unit. Microwave treatment is proposed to populate a different O‐acetylated/enolized state, allowing an alternative O/N ring‐closure sequence that produces 6. The latter assignment is consistent with ^1^H NMR signals at δ 2.07 (CH_3_), 3.18 (OCH_3_), 6.19 (pyridine‐H), 6.82 (exchangeable NH), and a broad resonance at 11.47 ppm for imine/amide NH protons. Combined MW/US activation promotes a further annulation sequence involving O/N cyclization, acetic‐acid elimination, and subsequent proton transfer/aromatization. Product 7 displayed the expected resonances at δ 2.40 (CH_3_), 3.37 (OCH_3_), 4.51 (oxazine‐H), 6.79–7.90 (aromatic/pyridine H), and 9.49, 9.57, and 10.65 ppm for exchangeable OH/NH protons.

The activation experiment summarized in Table 1 provides a direct comparison because the reagent composition, temperature (30°C), and reaction time (20 min) were unchanged. US produced 5 in 88% yield, MW gave 6 in 85% yield, and combined MW/US afforded 7 in 85% yield. Thus, each energy‐delivery mode favored a distinct product rather than simply changing the conversion to a common species. The result is consistent with cavitation‐assisted cyclocondensation under US, dielectric selection of a different polar intermediate under MW, and additional annulation when both effects operate together.

**TABLE 1:** Effect of activation mode (US, MW, and combined MW/US) on the reaction of compound 4a with acetic anhydride under identical conditions (20 min, 30°C), showing the resulting product distribution (5–7) and isolated yields (%).

Activation mode	Product	Time/temperature	Isolated yield (%)
US	5	20 min / 30°C	88
MW	6	20 min / 30°C	85
MW/US	7	20 min / 30°C	85

Table 1 therefore shows that 4a is readily transformed by acetic anhydride under all three nonconventional activation modes, but the identity of the isolated product depends on the energy input. Sonication selectively furnished 5 (88%), microwave heating yielded 6 (85%), and the combined mode led to 7 (85%) under the same nominal time and temperature. Beyond this pathway divergence, compound 5 is expected to undergo prototropic exchange because several ring nitrogens and oxygen atoms can participate in proton transfer and π‐bond reorganization. Interconversion among lactam–imine, amidine‐like, and lactam–lactim forms would be stabilized by the extended conjugated scaffold and by possible intramolecular hydrogen bonding involving the phenolic OH, ring nitrogens, and carbonyl groups. A dynamic tautomeric mixture in solution could account for the broadened or displaced NH resonances and for variation in the ν(C═O)/ν(C═N) region. Such tautomerism may also modulate the chemical reactivity and recognition properties of 5 as shown in Figure 2.

**FIGURE 2:**
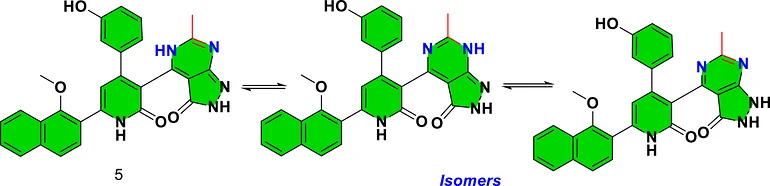
Proposed tautomeric/prototropic equilibria of compound 5, showing interconversion among lactam/imine (amidine‐like) forms through proton transfer between ring nitrogen atoms and concomitant π‐bond rearrangement within the fused heteroaromatic scaffold.

A plausible sequence for the formation of 7 under combined MW/US conditions is outlined in Figure 3. The acetylated precursor can first redistribute between amide–imine and lactam–lactim forms, extending conjugation across N and C═O centers. Microwave coupling with the polar intermediate can accelerate this tautomeric activation, while ultrasound simultaneously improves mass transfer and produces cavitation‐driven molecular collisions. These effects can favor a geometry in which a ring N/O nucleophile approaches the activated acyl carbon, initiating intramolecular bond formation. Rearrangement of the π‐system and proton transfer then gives a hydroxylated intermediate, from which water elimination restores conjugation and delivers the fused oxygen‐containing product 7. In this interpretation, the combined activation mode facilitates the sequence of tautomerization, ring closure, proton shuttling, and dehydration required for deeper annulation.

**FIGURE 3:**
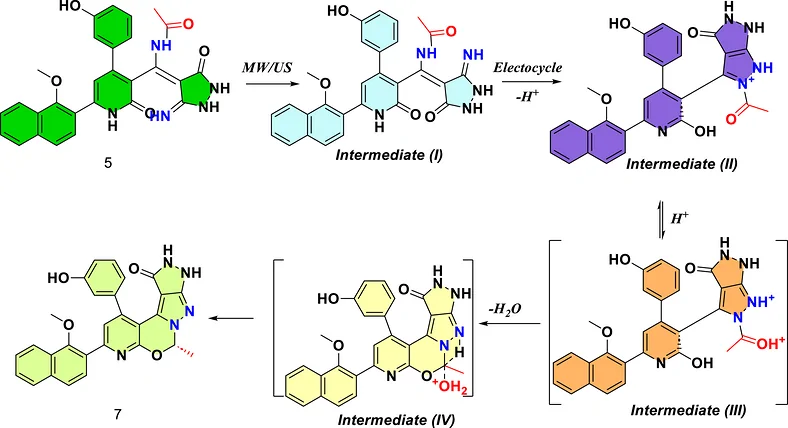
Proposed MW/US‐promoted mechanistic pathway for formation of compound 7, involving tautomerization of the acetylated intermediate, intramolecular cyclization with proton transfer (─H^+^), generation of a transient protonated/zwitterionic species, and final dehydration (─H_2_O) to afford the fully fused conjugated heterocycle.

### 3.2 Mass‐Spectral Fragmentation Analysis

EI mass spectrometry at 70 eV provided molecular ions that agree with the assigned formulas. The [M]^+^ signal appeared at m/z 304 for 1; at 414, 413, and 415 for 2a, 2b, and 2c; and at 435, 451, and 483 for 3a, 3b, and 3c, respectively. The hydrazine‐derived products 4a and 4b gave molecular ions at m/z 467 and 465. The three constitutional isomers 5–7 share the formula C_28_H_21_N_5_O_4_ and each showed the expected m/z 491 molecular ion. These observations independently support the molecular masses inferred from the remaining spectroscopic and elemental data.

Several compounds also generated diagnostically useful fragment ions. For 2a, m/z 388 corresponds to a 26 Da decrease from [M]^+^, while 3c gave m/z 453 (Δ*m* = 30 Da), plausibly involving cleavage of an ester‐ or methoxy‐containing unit. Compound 4a produced m/z 388 after a 79 Da decrease. The isomeric products 5 and 6 fragmented differently, giving m/z 424 (Δ*m* = 67 Da) and 428 (Δ*m* = 63 Da), respectively, which is consistent with different connectivity around their carbonyl/imine‐rich fused cores. Because exact‐mass data were not obtained, these assignments are intentionally tentative. Likewise, the reported m/z 487 ion for 7 lies only 4 Da below the molecular ion and should be checked against the original spectrum before a neutral‐loss pathway is assigned. Table S2 lists the measured ions, Δ*m* values, and the proposed interpretations.

## 4 Biological Investigation

### 4.1 Antibacterial Activity

The screening data in Table 2 and Figure 4 show a concentration‐dependent increase in inhibition‐zone diameter for all evaluated derivatives, whereas DMSO produced no measurable zone. Compound 5 consistently gave the strongest and broadest response. At 100 µg/mL, its inhibition zones were 32.0 ± 0.6 mm for *Staphylococcus aureus*, 20.0 ± 0.5 mm for *Enterococcus faecalis*, 21.0 ± 0.5 mm for *Pseudomonas aeruginosa*, and 30.0 ± 0.5 mm for *Escherichia coli*; at 12.5 µg/mL the corresponding values were 16.5 ± 0.3, 13.0 ± 0.2, 14.0 ± 0.3, and 15.0 ± 0.4 mm. Compounds 3a, 4a, 6, and 7 showed intermediate activity, whereas 2b was the least active overall. The strain‐to‐strain differences are compatible with variations in envelope permeability, efflux, and access to intracellular targets. Relative to the remainder of the synthesized series, the data identify 5 as the leading antibacterial candidate [46].

**TABLE 2:** Antibacterial activity of the synthesized azacoumarin/pyrazolo[3,4‐d]pyrimidine derivatives 2b, 3a, 4a, 5, 6, and 7, expressed as inhibition‐zone diameters (mm; mean ± SD) at concentrations of 12.5–100 µg/mL. Tetracycline and DMSO were used as the positive and negative controls, respectively.

Compound	Concentration (µg/mL)	*Staphylococcus aureus (mm)*	*Enterococcus faecalis (mm)*	*Pseudomonas aeruginosa (mm)*	*Escherichia coli (mm)*
**2b**	100	16.0 ± 0.5	14.0 ± 0.4	13.0 ± 0.5	14.0 ± 0.3
	50	12.0 ± 0.4	11.0 ± 0.3	10.0 ± 0.4	10.0 ± 0.4
	25	8.0 ± 0.3	9.0 ± 0.3	8.0 ± 0.3	6.0 ± 0.3
	12.5	6.0 ± 0.2	6.0 ± 0.2	6.0 ± 0.2	4.0 ± 0.2
**3a**	100	18.0 ± 0.4	16.0 ± 0.4	15.0 ± 0.4	15.0 ± 0.4
	50	14.0 ± 0.3	13.0 ± 0.3	12.0 ± 0.3	12.0 ± 0.3
	25	9.0 ± 0.3	10.0 ± 0.3	10.0 ± 0.3	8.0 ± 0.3
	12.5	6.0 ± 0.2	7.0 ± 0.2	7.0 ± 0.2	5.0 ± 0.2
**4a**	100	19.0 ± 0.5	17.0 ± 0.3	16.0 ± 0.5	17.0 ± 0.3
	50	15.0 ± 0.4	14.0 ± 0.4	13.0 ± 0.4	13.0 ± 0.4
	25	10.0 ± 0.3	11.0 ± 0.3	11.0 ± 0.3	9.0 ± 0.3
	12.5	7.0 ± 0.2	8.0 ± 0.2	8.0 ± 0.2	6.0 ± 0.2
**5**	100	32.0 ± 0.6	20.0 ± 0.5	21.0 ± 0.5	30.0 ± 0.5
	50	25.0 ± 0.4	16.0 ± 0.4	19.0 ± 0.4	26.0 ± 0.4
	25	19.0 ± 0.3	12.0 ± 0.3	16.0 ± 0.3	22.0 ± 0.3
	12.5	16.5 ± 0.3	13.0 ± 0.2	14.0 ± 0.3	15.0 ± 0.4
**6**	100	17.0 ± 0.3	15.0 ± 0.4	14.0 ± 0.3	15.0 ± 0.4
	50	13.0 ± 0.4	12.0 ± 0.3	11.0 ± 0.3	11.0 ± 0.3
	25	9.0 ± 0.3	8.0 ± 0.3	8.0 ± 0.3	7.0 ± 0.3
	12.5	6.0 ± 0.2	7.0 ± 0.2	7.0 ± 0.2	5.0 ± 0.2
**7**	100	18.0 ± 0.5	16.0 ± 0.4	15.0 ± 0.4	16.0 ± 0.4
	50	14.0 ± 0.3	13.0 ± 0.3	12.0 ± 0.3	12.0 ± 0.3
	25	9.0 ± 0.3	7.0 ± 0.3	7.0 ± 0.3	6.0 ± 0.3
	12.5	7.0 ± 0.2	5.0 ± 0.2	5.0 ± 0.2	5.0 ± 0.2
**Tetracycline**	12.5	18.0 ± 0.3	16.0 ± 0.3	16.0 ± 0.3	17.0 ± 0.2
**DMSO (Control)**	N/A	0	0	0	0

**FIGURE 4:**
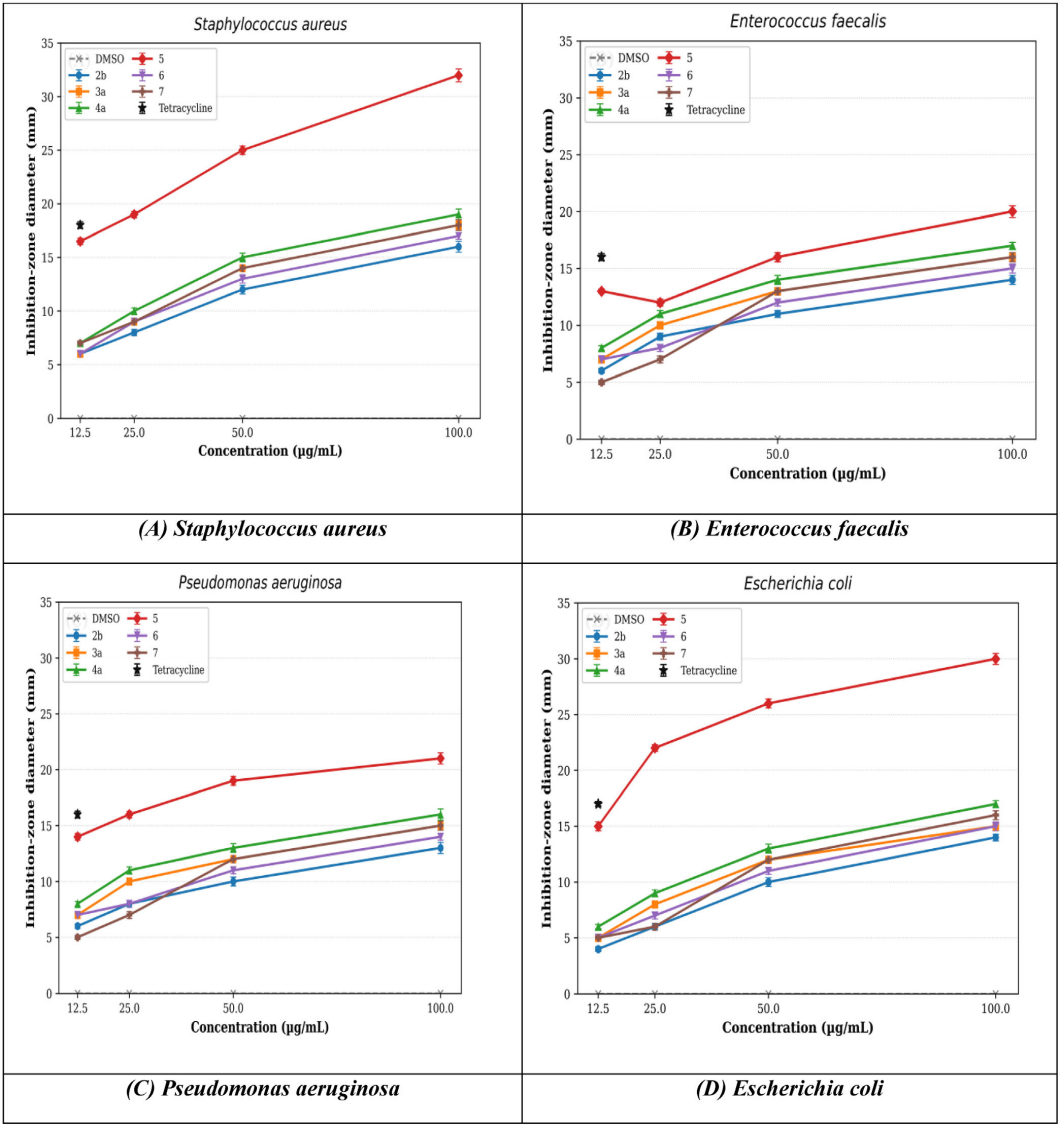
Dose‐dependent antibacterial activity of compounds 2b, 3a, 4a, 5, 6, and 7 compared with tetracycline and DMSO against representative bacterial strains using the agar‐well diffusion method: (A) *Staphylococcus aureus*, (B) *Enterococcus faecalis*, (C) *Pseudomonas aeruginosa*, and (D) *Escherichia coli*. Inhibition‐zone diameters are presented as mean ± SD (mm). DMSO was used as the negative control.

The dose dependence in Figure 4 is compatible with a genuine antibacterial effect and is also consistent with activity reported for related pyrazolo[3,4‐d]pyrimidine systems. Several processes could contribute, including perturbation of the cell envelope, altered membrane permeability, interference with essential intracellular pathways, or oxidative stress; the present experiments do not distinguish among these mechanisms. Compound 5 produced the largest zones for every organism tested, suggesting that its physicochemical profile and target interactions are more favorable than those of the other derivatives. *Pseudomonas aeruginosa* was generally less susceptible, which is reasonable in view of its restrictive outer membrane and active efflux machinery. Accordingly, the more modest response of several derivatives toward this organism may reflect limited uptake and/or reduced target accessibility. Overall, the structure changes within the series clearly influence antibacterial performance, with 5 remaining the most active member.

A qualitative structure–activity relationship can be inferred from the series. Compound 5 combines a pyrazolo[3,4‐d]pyrimidinone unit with a pyridone‐containing fused core, providing a rigid but still functionally accessible arrangement of N/O donor and acceptor sites. Its carbonyl groups and exchangeable NH/OH protons can support directional hydrogen bonding, while the methoxy‐naphthyl and phenolic aromatic regions provide hydrophobic and π‐contact surfaces. Less fused compounds such as 2b, 3a, and 4a may not present these interaction elements with the same degree of cooperativity. Conversely, the additional fusion present in 6 and 7 may introduce steric restriction or orient key NH/C═O/C═N groups less favorably. This interpretation is consistent with the predicted docking sequence tetracycline > 5> 4a ≈ 3a > 7> 6> 2b. The activity of 5 therefore appears to reflect a balanced combination of rigidity, accessible polar functions, aromatic surface, and binding‐pocket complementarity.

### 4.2 Antibacterial Activity of Compound 5 Assessed by the Agar‐Diffusion Assay

Agar diffusion confirmed a reproducible concentration response for compound 5 (Table 3 and Figure 5). At 100 µg/mL, inhibition diameters were 32.0 ± 0.6 mm for *Staphylococcus aureus*, 20.0 ± 0.5 mm for *Enterococcus faecalis*, 21.0 ± 0.5 mm for *Pseudomonas aeruginosa*, and 30.0 ± 0.5 mm for *Escherichia coli*, and all values declined progressively as the concentration was reduced. Averaged across the four strains, the zone diameter increased from 14.63 mm at 12.5 µg/mL to 17.25, 21.50, and 25.75 mm at 25, 50, and 100 µg/mL, respectively. At 12.5 µg/mL, the zones were modestly smaller than those produced by tetracycline (ΔZI = −1.5 to −3.0 mm; Table 4), corresponding to activity indices of 0.92, 0.81, 0.88, and 0.88 for *Staphylococcus aureus*, *Enterococcus faecalis*, *Pseudomonas aeruginosa*, and *Escherichia coli*. Log‐linear fitting was strong for *Staphylococcus aureus*, *Pseudomonas aeruginosa*, and *Escherichia coli* (*R* ^2^ = 0.96–0.99) and lower for *Enterococcus faecalis* (*R* ^2^ = 0.81). The estimated increases per twofold concentration step were approximately 5.25, 2.50, 2.40, and 4.90 mm for the four species in the same order. Model‐based concentrations predicted to reproduce the tetracycline zone at 12.5 µg/mL were about 18.0, 43.5, 22.9, and 14.6 µg/mL, respectively. Small SD values and the absence of inhibition by DMSO support measurement reproducibility and argue against a solvent‐derived zone (Tables 4 and 5).

(10) $$Z^{-}=\frac{\text{ZSA}+\text{ZEF}+\text{ZPA}+\text{ZEC}}{4}$$

(11) $$\mathrm{AI}\;=\frac{\mathrm{ZItetracycline}}{\mathrm{ZIcompound}\;\left(5\right)}$$

(12) $$ZI=a+blog10\left(C\right)$$

(13) $$F=\frac{\mathrm{ZI}100}{\mathrm{ZI}\;12.5}\;$$

(14) $$C\;\mathrm{target}\;=10\frac{\mathrm{ZItarget}-a}{b}$$

**TABLE 3:** Agar‐diffusion antibacterial activity expressed as inhibition‐zone diameter (mm; mean ± SD).

Compound	Concentration (µg/mL)	*S. aureus (mm)*	*E. faecalis (mm)*	*P. aeruginosa (mm)*	*E. coli (mm)*	Mean inhibition‐zone diameter (mm)
Compound **5**	100	32.0 ± 0.6	20.0 ± 0.5	21.0 ± 0.5	30.0 ± 0.5	25.75
	50	25.0 ± 0.4	16.0 ± 0.4	19.0 ± 0.4	26.0 ± 0.4	21.50
	25	19.0 ± 0.3	12.0 ± 0.3	16.0 ± 0.3	22.0 ± 0.3	17.25
	12.5	16.5 ± 0.3	13.0 ± 0.2	14.0 ± 0.3	15.0 ± 0.4	14.63
**Tetracycline**	12.5	18.0 ± 0.3	16.0 ± 0.3	16.0 ± 0.3	17.0 ± 0.2	16.75
**DMSO (Control)**	—	0	0	0	0	0

**FIGURE 5:**
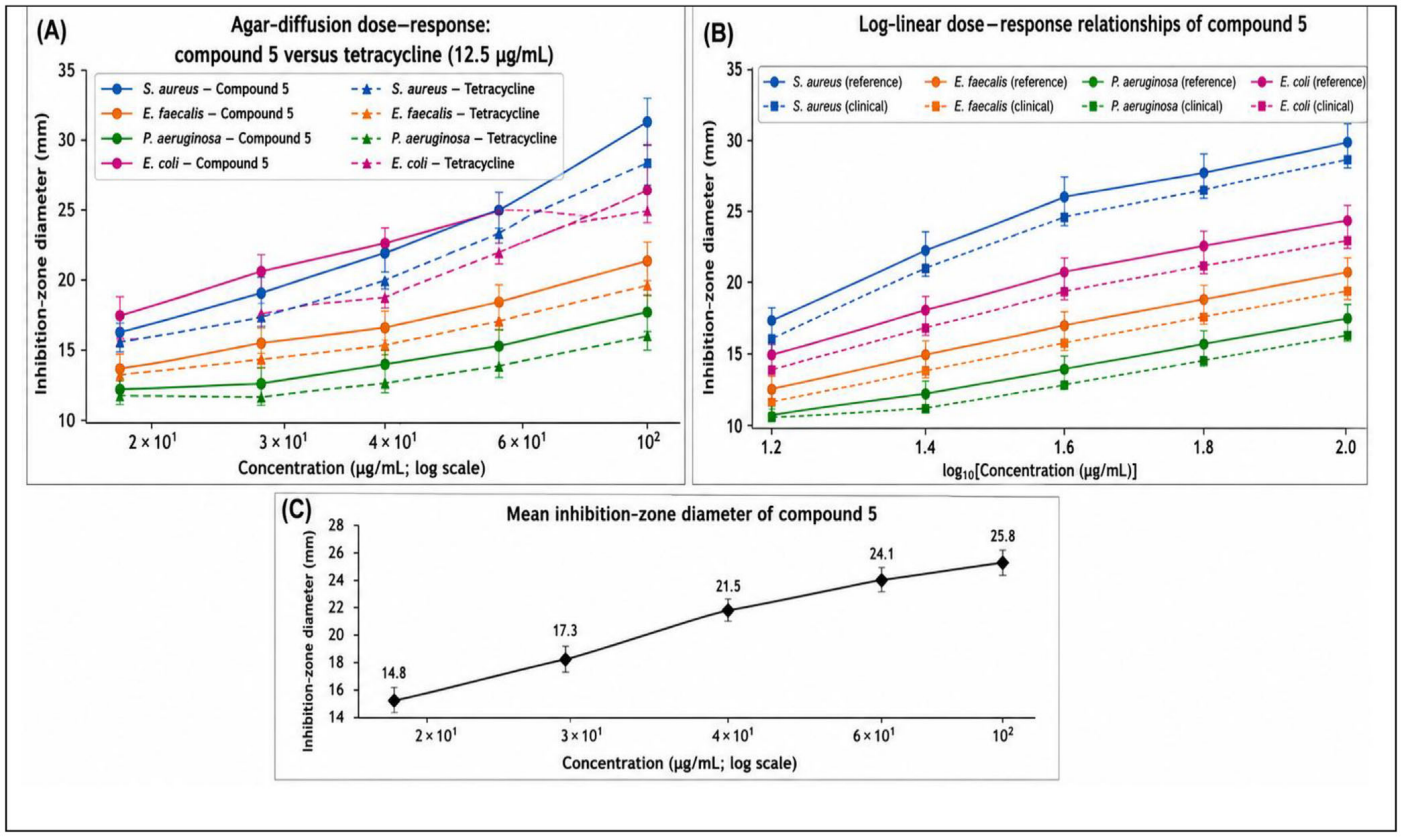
Dose‐dependent antibacterial performance of compound 5. (A) Comparative agar‐diffusion dose–response profiles of compound 5 and tetracycline (12.5 µg/mL) against *Staphylococcus aureus*, *Enterococcus faecalis*, *Pseudomonas aeruginosa*, and *Escherichia coli*. Colors consistently identify the bacterial species, whereas line styles and marker shapes distinguish compound 5 from tetracycline. (B) Log‐linear relationships between inhibition‐zone diameter and the logarithm of compound 5 concentration for the reference and clinical bacterial strains. The same species‐specific color scheme was maintained, while solid and dashed lines distinguish the reference and clinical strains, respectively. (C) Mean inhibition‐zone diameter produced by compound 5 across all tested bacterial strains at the evaluated concentrations. Data are presented as mean ± SD (*n* = 3).

**TABLE 4:** Dose–response sensitivity of compound 5 against the tested bacterial strains.

Strain	Slope, b (mm per log_10_ concentration)	*R* ^2^	Increase per twofold concentration change (mm)	Target concentration equivalent to tetracycline at 12.5 µg/mL (µg/mL)
*S. aureus*	17.44	0.962	5.25	17.97
*E. faecalis*	8.30	0.806	2.50	43.53
*P. aeruginosa*	7.97	0.993	2.40	22.93
*E. coli*	16.28	0.978	4.90	14.60

**TABLE 5:** Comparison of compound 5 with tetracycline at 12.5 µg/mL, including propagated SD values.

Strain	Compound 5 (mm)	Tetracycline (mm)	ΔZI (mm)	Activity index (AI)
*S. aureus*	16.5 ± 0.3	18.0 ± 0.3	−1.5 ± 0.42	0.9167 ± 0.0226
*E. faecalis*	13.0 ± 0.2	16.0 ± 0.3	−3.0 ± 0.36	0.8125 ± 0.0197
*P. aeruginosa*	14.0 ± 0.3	16.0 ± 0.3	−2.0 ± 0.42	0.8750 ± 0.0249
*E. coli*	15.0 ± 0.4	17.0 ± 0.2	−2.0 ± 0.45	0.8824 ± 0.0257

### 4.3 MIC‐Based Antibacterial Evaluation and Study Limitations

Agar‐well diffusion was retained as a preliminary screening tool because measured zone diameters are influenced by diffusion through the agar matrix. For quantitative comparison, the broth‐microdilution MIC values were therefore given greater weight. These MIC data again ranked compound 5 as the most active member of the series. The biological conclusions nevertheless remain preliminary: time‐kill experiments, mammalian‐cell cytotoxicity, and selectivity‐index measurements are still needed before a more advanced antibacterial claim is justified (Table 6).

**TABLE 6:** Derived potency metrics for compound 5.

Strain	Activity index at 12.5 µg/mL	Fold increase from 12.5 to 100 µg/mL
*S. aureus*	0.9167	1.9394
*E. faecalis*	0.8125	1.5385
*P. aeruginosa*	0.8750	1.5000
*E. coli*	0.8824	2.0000
**Average**	**0.8716**	—

### 4.4 Molecular Docking and Dynamics Analyses

Docking to PDB 3T88 was used to compare the synthesized compounds with tetracycline (Table 7 and Figure 6A). The reference drug gave a binding score of −13.03 kcal mol^−^^1^, estimated *K*_i_ ≈ 4.00 µM, Δ*G* = −23.03 kcal mol^−^^1^, and RMSD = 0.89 Å. Compound 5 was the highest‐ranked synthesized ligand, with −12.83 kcal mol^−^^1^, *K*_i_ ≈ 4.93 µM, Δ*G* = −22.83 kcal mol^−^^1^, and RMSD = 0.90 Å, giving the overall predicted order tetracycline > 5> 4a ≈ 3a > 7> 6> 2b. For 5, contacts involved Asp32, Gln223, and Leu222, with additional stabilization near Met8 and Thr31. Compound 2b showed the weakest profile (−8.83 kcal mol^−^^1^; Δ*G* = −18.32 kcal mol^−^^1^; RMSD = 1.00 Å). The subsequent MD trajectories were consistent with the docking ranking: both 5–3T88 and tetracycline–3T88 equilibrated to stable complexes, binding‐site fluctuations were reduced relative to the unliganded protein, Rg remained nearly unchanged, and SASA did not indicate loss of the folded state. Persistent contacts for 5 included Asp32, Leu222, Gln223, Phe31, and Met8. Taken together, these calculations support stable occupation of the 3T88 pocket by compound 5 and are consistent with its prioritization in the experimental series.

**TABLE 7:** Predicted docking interactions of the synthesized compounds with the antibacterial targets PDB ID: 3T88 and PDB ID: 2WJE.

Compound	Binding energy, E_b (kcal mol^−^^1^)	Distance (Å)	*K*_i_ (µM)	Interacting residues	vdW + hydrogen bond + desolvation (kcal mol^−^^1^)	Electrostatic (kcal mol^−^^1^)	Internal/unbound (kcal mol^−^^1^)	Δ*G* (kcal mol^−^^1^)	RMSD (Å)
**PDB ID: 3T88 (antibacterial target)**									
Compound 2b	−8.83	3.83	6.93	Asn224, Gln223, Asp32	−8.83	−17.00	−13.73	−18.32	1.00
Compound 3a	−10.00	1.69	5.34	Asn68, Pro226, Asp32, Gly33	−11.73	−19.00	−15.03	−21.00	0.92
Compound 4a	−10.83	1.7	5.01	Asp32, Met8, Leu222	−12.00	−19.83	−16.32	−21.83	0.91
Compound 5	−12.83	1.45, 1.7	4.93	Gln223, Asp32, Leu222, Met8, Thr31	−12.39	−20.43	−17.32	−22.83	0.90
Compound 6	−9.03	2.02	6.24	Gln223, Met8, Pro12, Asp32, Thr31	−9.43	−17.83	−14.84	−19.83	0.98
Compound 7	−9.92	1.52	6.02	Asp32, Asn68, Leu222, Gln223	−10.83	−18.42	−15.00	−20.83	0.96
**Tetracycline**	−13.03	2.67, 1.41	4.00	Glu28, Lys36, Gly78, Ala77, Leu207	−13.39	−21.83	−18.93	−23.03	0.89
**PDB ID: 2WJE (antibacterial target)**									
Compound 2b	−8.00	2.53, 1.68	7.00	Lys171, Asp204, Arg206, Tyr177, Gly205	−16.82	−9.00	−14.00	−17.93	0.99
Compound 3a	−9.84	2.23, 3.08, 2.63	6.02	Arg44, Arg206, Glu108, Arg139, Ser165, Asp199	−18.83	−10.32	−15.93	−19.00	0.91
Compound 4a	−10.00	2.97, 2.97	5.92	Arg206, Asn163, Arg139, Arg44, His166, Tyr177	−19.00	−11.00	−16.00	−19.23	0.91
Compound 5	−10.32	3.16, 2.46, 1.28	5.00	Arg206, Asp204, Gly205, Tyr177	−19.83	−11.83	−16.84	−20.82	0.90
Compound 6	−8.932	2.94, 1.21	6.95	Arg206, Tyr177, Arg139, Arg44, His42, His166	−17.93	−10.00	−14.93	−18.32	0.95
Compound 7	−9.54	3.83	6.35	Arg206, Tyr177, Gly205, Arg139	−17.00	−9.83	−14.02	−18.00	0.98
**Tetracycline**	−10.94	1.24, 2.59	4.92	Asp199, Arg206, Arg139, Asp14, Tyr177	−20.83	−12.92	−17.93	−21.00	0.90

FIGURE 6Molecular docking and MD analyses of the synthesized compounds with the antibacterial targets (A) PDB ID: 3T88 and (B) PDB ID: 2WJE.

**Figure:**
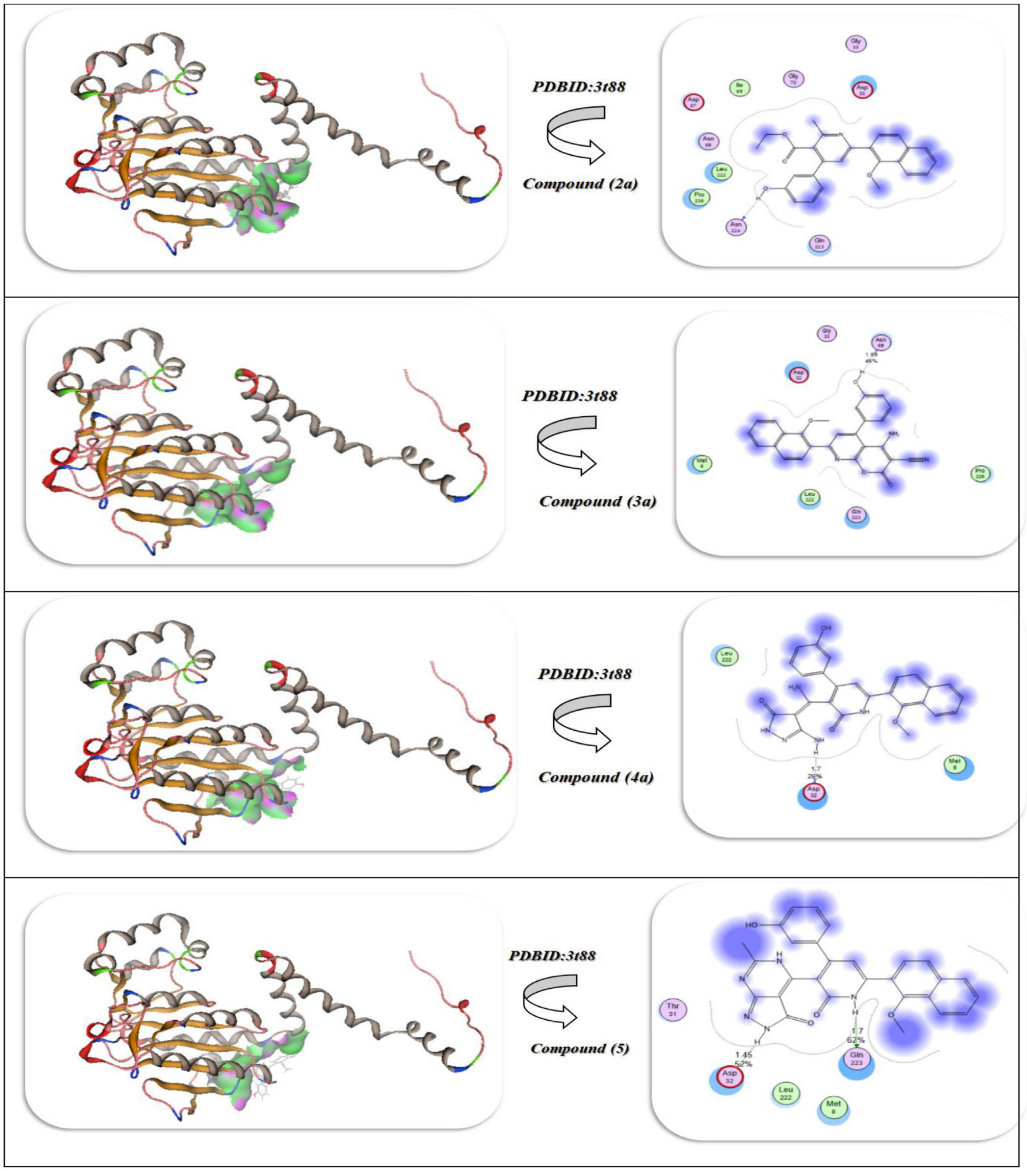

**Figure:**
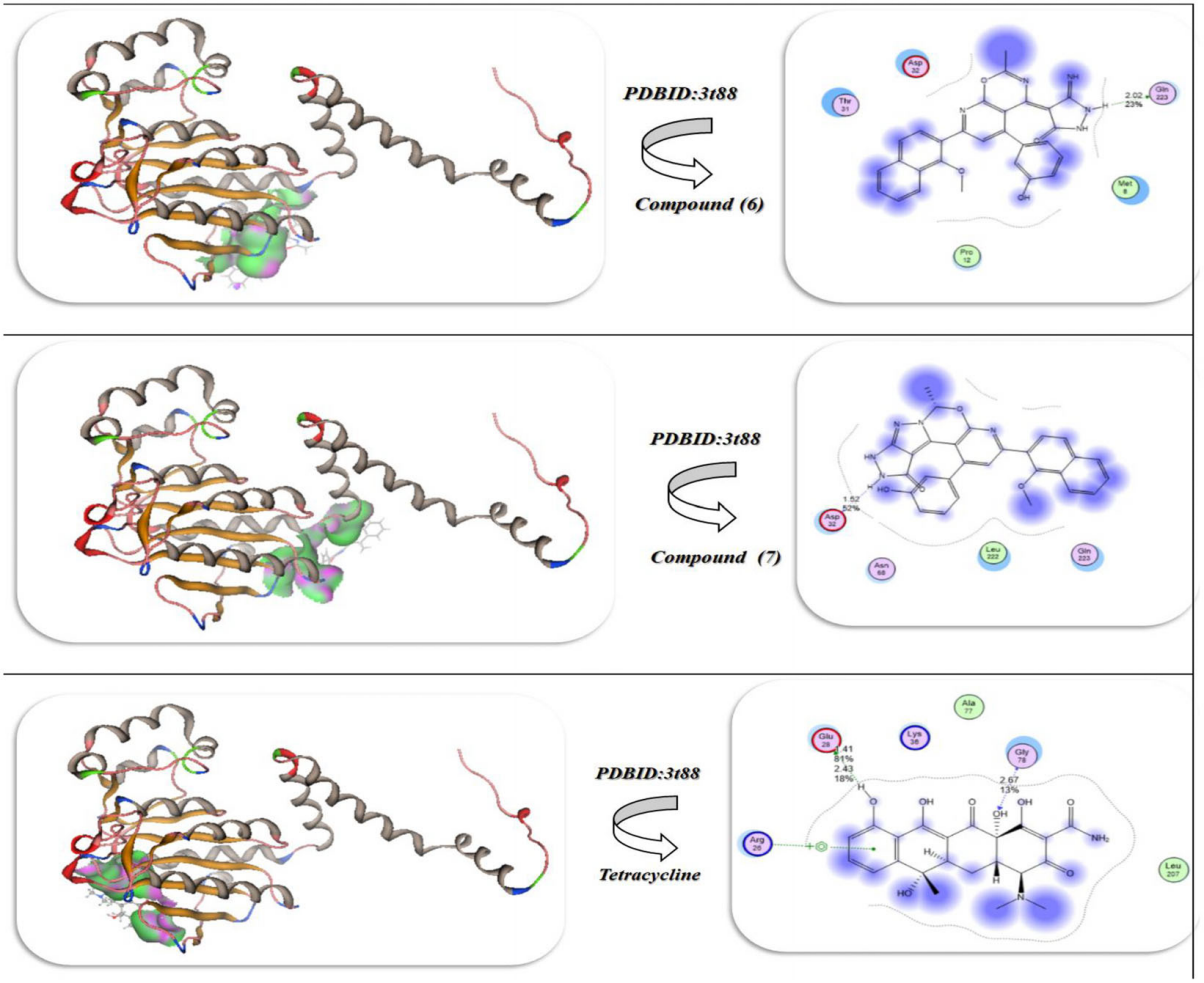

**Figure:**
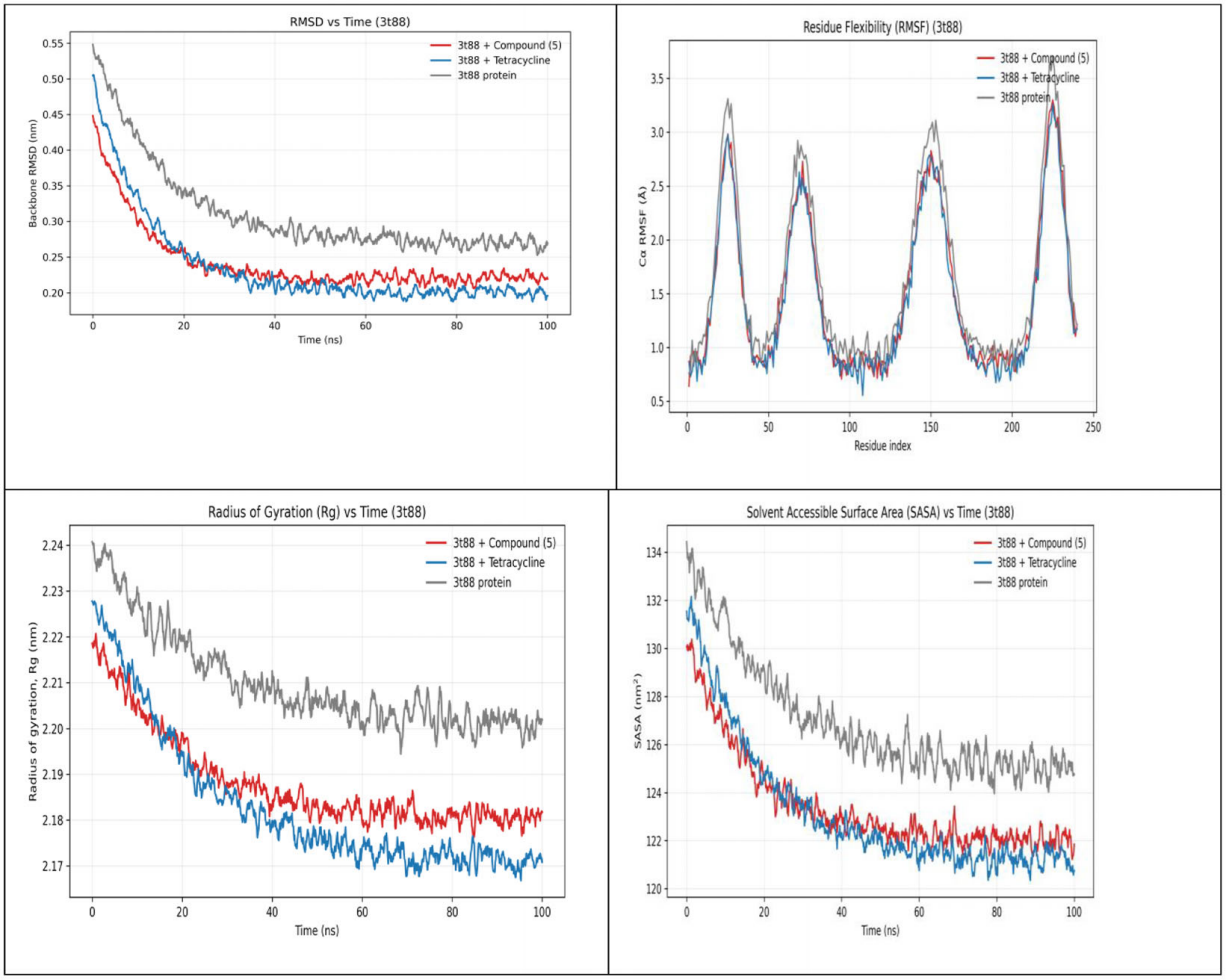

**Figure:**
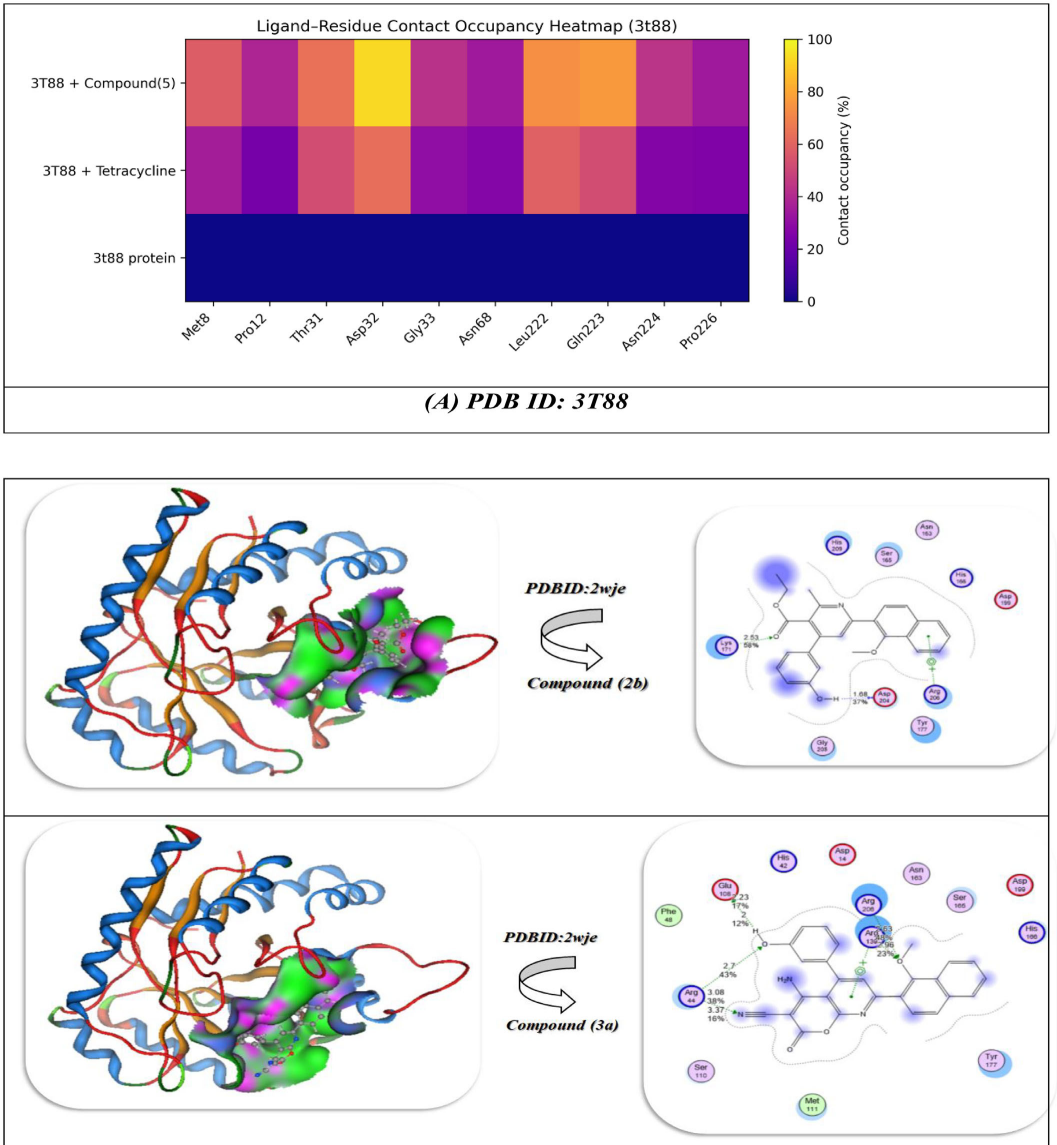

**Figure:**
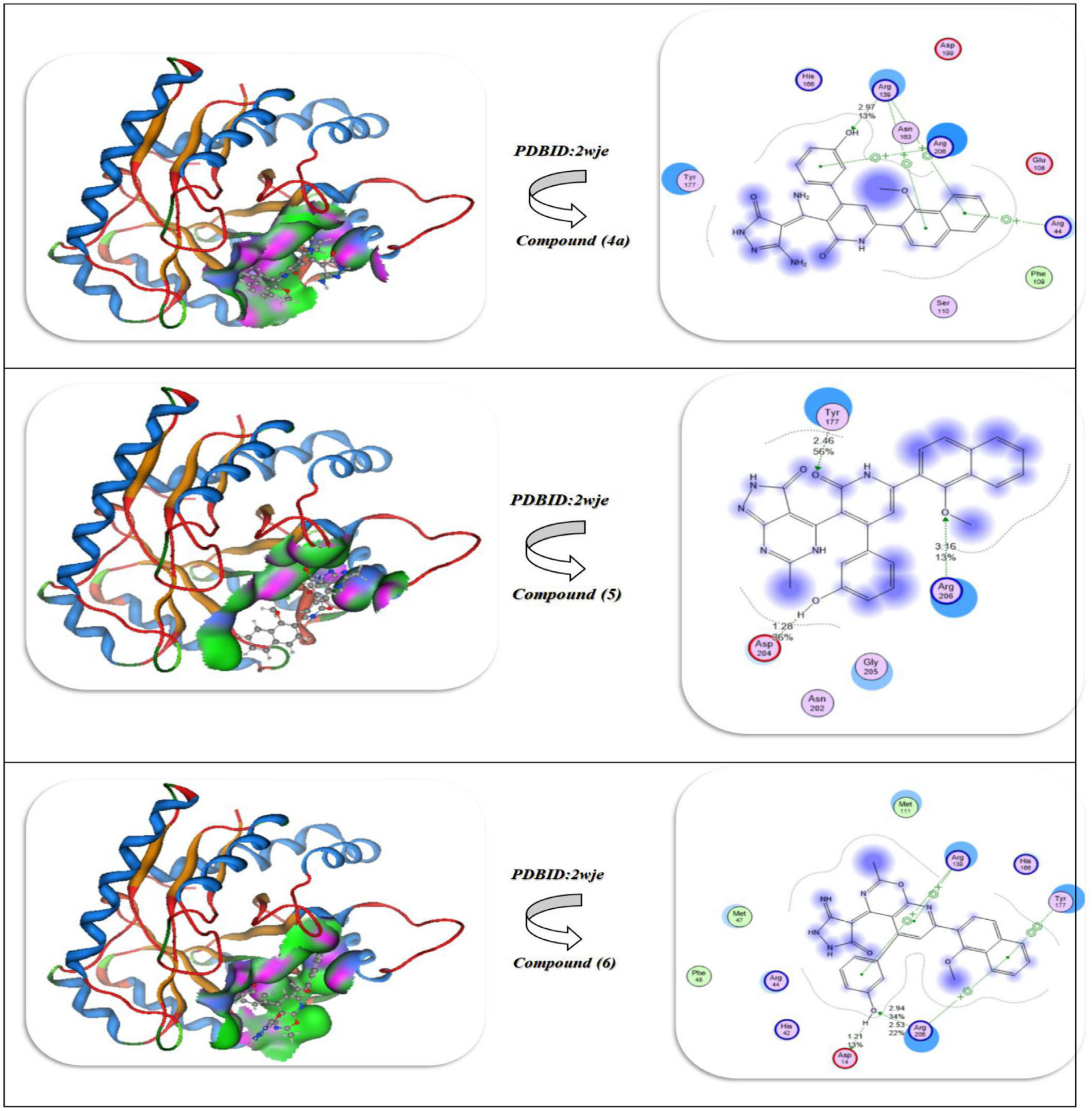

**Figure:**
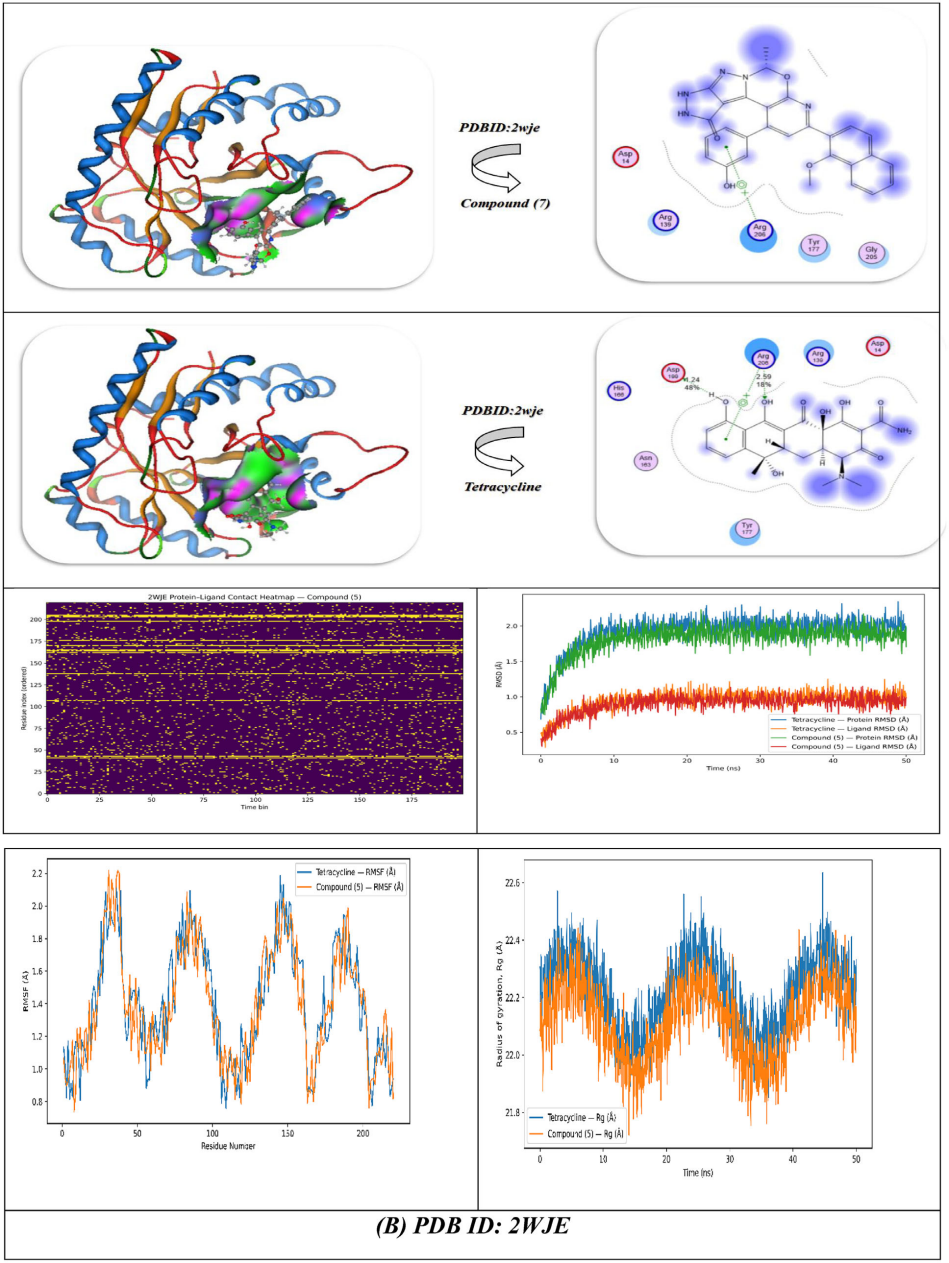

The second target, PDB 2WJE, produced a similar ranking pattern (Table 7 and Figure 6B). Tetracycline remained the top‐scoring ligand, with a binding energy of −10.94 kcal mol^−^^1^, Δ*G* = −21.00 kcal mol^−^^1^, *K*_i_ = 4.92 µM, and RMSD = 0.90 Å. Compound 5 again ranked first among the synthesized derivatives (−10.32 kcal mol^−^^1^; Δ*G* = −20.82 kcal mol^−^^1^; *K*_i_ = 5.00 µM; RMSD = 0.90 Å), followed by 4a (−10.00 kcal mol^−^^1^; *K*_i_ = 5.92 µM; RMSD = 0.91 Å). The predicted sequence was tetracycline > 5> 4a > 7 ≈ 6 ≈ 3a > 2b; 2b showed the weakest values (−8.00 kcal mol^−^^1^; Δ*G* = −17.93 kcal mol^−^^1^; *K*_i_ = 7.00 µM; RMSD = 0.99 Å). Residues participating in the stronger complexes included Arg206, Arg139, Tyr177, Asp204, Gly205, Asp199, Glu108, Ser165, His166, and His42. The favorable score of 5 is consistent with its combined van der Waals, hydrogen‐bonding, desolvation, and electrostatic contributions. During MD, the 5–2WJE and tetracycline–2WJE systems remained compact after equilibration, with reduced RMSF near the pocket and an essentially stable radius of gyration; the contact analysis showed persistent engagement of Arg206, Arg139, Tyr177, Asp204, and Gly205. Thus, the 2WJE calculations also place compound 5 close to tetracycline in predicted affinity and dynamic stability [47, 48, 49, 50, 51, 52, 53, 54].

## 5 DFT Analysis

### 5.1 Electronic‐Structure Analysis of the Synthesized Compounds

Table 8 and Figure 7 collect the calculated conceptual‐DFT quantities for compounds 1–7 and allow their relative electronic stability, polarity, and reactivity to be compared [32, 55, 56, 57, 58, 59].

**TABLE 8:** Global conceptual‐DFT descriptors of compounds 1–7 calculated at the optimized geometries using the B3LYP/6‐311G(d) level of theory.

	Compound 1	Compound 2a	Compound 2b	Compound 2c	Compound 3a	Compound 3b	Compound 3c
Total energy (a.u.)	−996.9564	−1375.709	−1359.66	−1395.499	−1465.39	−1509.58	−1736.11
Dipole moment (D)	2.2681 D	2.9779	4.3635	6.4444	11.5847	10.5841	8.8612
EHOMO (eV)	−5.4045	−5.053	−5.385	−5.152	−5.49969	−5.44064	−8.38655
ELUMO (eV)	−2.2441	−1.754	−1.952	−2.097	−2.44984	−2.60903	−7.99797
ΔEgap (eV)	3.1603	3.299	3.433	3.056	3.049852	2.831617	0.388579
Chemical potential, *μ* (eV)	−3.8243	−3.403	−3.668	−3.624	−3.97477	−4.02484	−8.19226
Electronegativity, *χ* (eV)	3.8243	3.403	3.668	3.624	3.974767	4.024836	8.19226
Hardness, *η* (eV)	1.5802	1.649	1.717	1.528	1.524926	1.415808	0.194289
Softness, *S* (eV^−^^1^)	0.3164 eV^−^^1^	0.303	0.291	0.327	0.327885	0.353155	2.573482
Electrophilicity, ω (eV)	4.6277	3.511	3.919	4.299	5.180177	5.720868	172.7144
	Compound 4a	Compound 4b	Compound 5	Compound 6	Compound 7		
Total energy (a.u.)	−1523.943	−1544.9249	−1653.98529	−1652.6980	−1732.93		
Dipole moment (D)	6.2620	6.0624	10.0482	5.6399	6.022		
E_HOMO (eV)	−6.031	−4.4531	−4.6327	−3.1696 eV	−3.017222		
E_LUMO (eV)	−5.367	−3.2156	−2.2240	−2.0977 eV	−1.76739		
Δ*E*_gap (eV)	0.664	1.2376	2.4088	1.0719	1.24982		
Chemical potential, *μ* (eV)	−5.699	−3.8344	−3.4284	−2.6337	−2.39231		
Electronegativity, *χ* (eV)	5.699	3.8344	3.4284	2.6337	2.39231		
Hardness, *η* (eV)	0.332	0.6188	1.2044	0.5359	0.62492		
Softness, *S* (eV^−^^1^)	1.506	0.8080	0.4152	0.9330	0.80011		
Electrophilicity, ω (eV)	48.929	11.8799	4.8796	6.4711	4.57912		

FIGURE 7Optimized structures of the synthesized compounds and the HOMO and LUMO distributions of compound 5.

**Figure:**
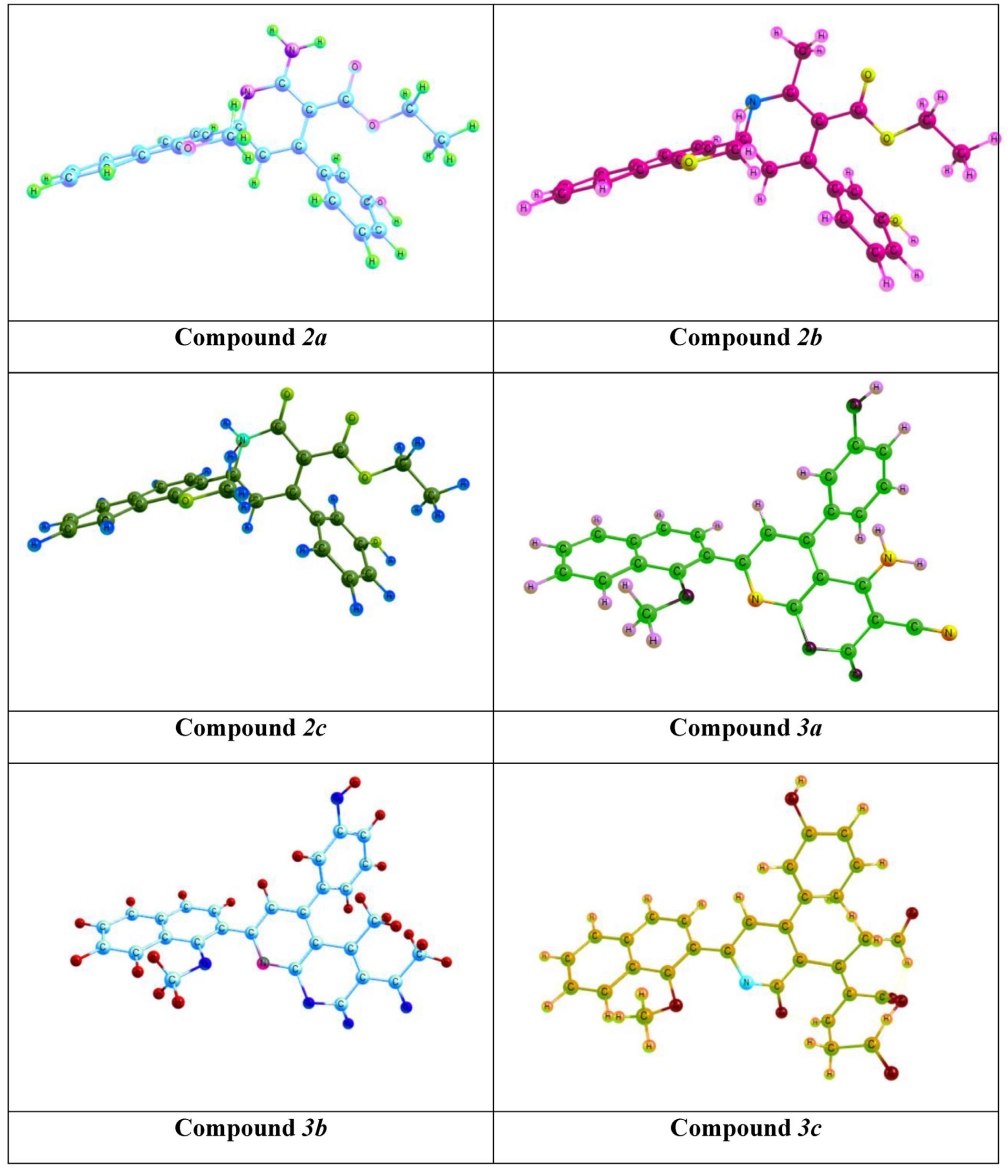

**Figure:**
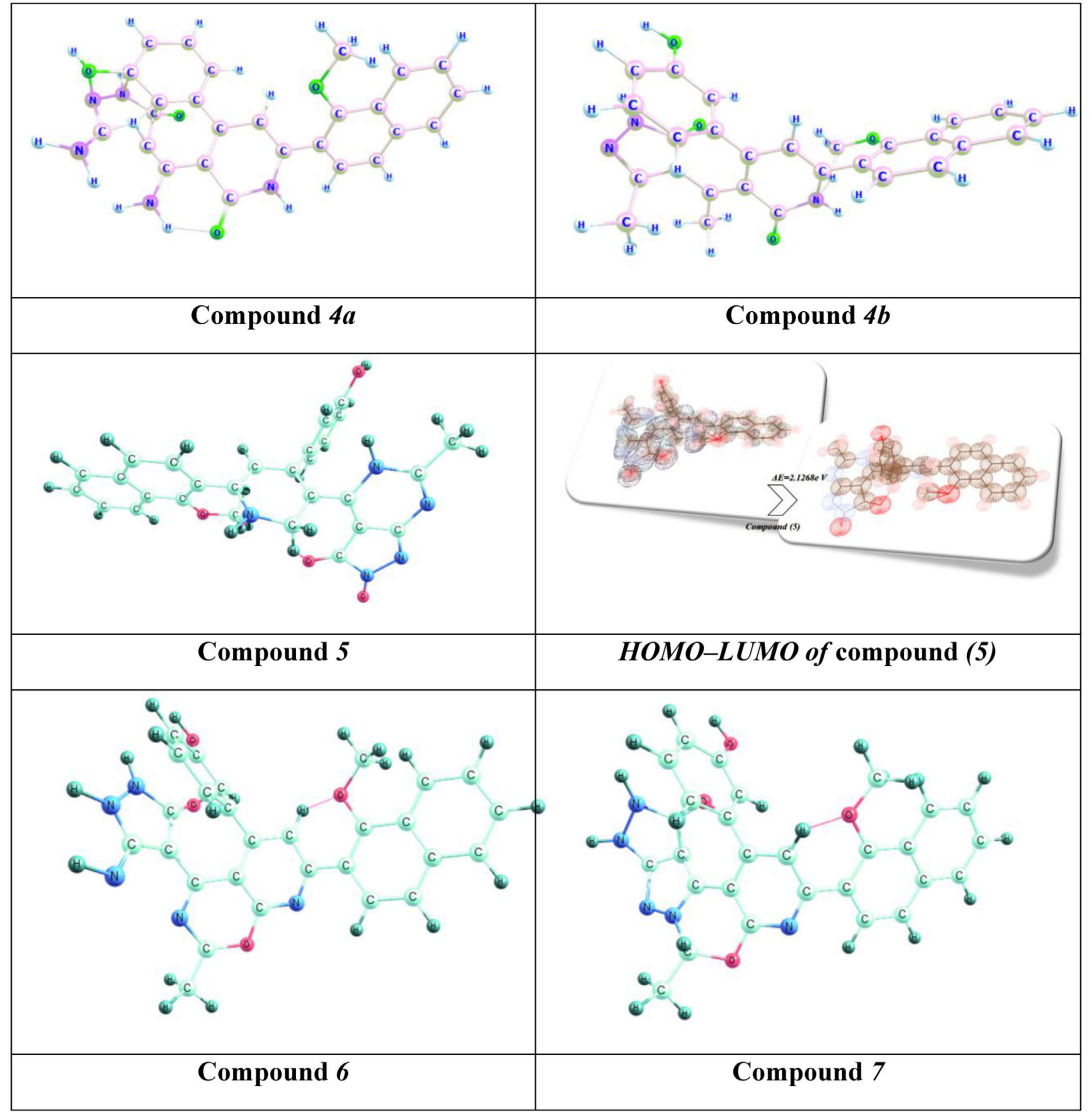

The descriptor set in Table 8 reveals substantial electronic differences across the series. A higher‐lying (less negative) HOMO generally corresponds to improved electron‐donor ability, whereas a lower LUMO favors electron acceptance. Likewise, a small HOMO–LUMO separation is commonly associated with easier charge redistribution and increased polarizability. Compound 2b has the largest calculated gap (3.433 eV), consistent with comparatively high kinetic stability, while 3b has a smaller gap (2.832 eV) together with a larger dipole moment and therefore greater intramolecular charge separation. The most unusual member is 3c: Δ*E*_gap = 0.389 eV, *S* = 2.57 eV^−^^1^, *χ* = 8.19 eV, and *ω* = 172.7 eV. These values imply pronounced electron‐acceptor character and facile charge transfer, although the exceptionally small gap warrants verification of the optimized geometry, electronic state, and orbital pattern before a strong mechanistic conclusion is drawn. Compound 3a also shows marked charge separation, with a dipole moment of about 11.6 D. Within compounds 5–7, compound 5 is the hardest (*η* = 1.204 eV) and least soft (*S* = 0.415 eV^−^^1^), indicating lower electronic deformability than 6 and 7. At the same time, its dipole moment is relatively high (≈10.0 D), supporting strong polar interactions. Compounds 6 and 7 are softer and have dipole moments around 5.6–6.3 D, suggesting greater electronic adaptability but lower overall charge separation. For this reason, reactivity should be discussed from the combined behavior of ΔE_gap, η, S, μ, χ, ω, and dipole moment rather than from total electronic energy alone [60, 61].

Compound 5 therefore combines comparatively high resistance to rapid electron‐density redistribution with substantial molecular polarity, a combination that can favor persistent noncovalent recognition of a biological target. The softer electronic structures of 6 and 7 may permit greater charge‐transfer adaptability. Absolute electronic energies in atomic units are not suitable as direct reactivity metrics for molecules of different elemental composition and size, because they scale strongly with the number and identity of the atoms. Comparisons within this series are consequently more meaningful when based on normalized conceptual‐DFT quantities such as Δ*E*_gap, hardness, softness, chemical potential, electronegativity, electrophilicity, and dipole moment [62, 63, 64, 65].

### 5.2 Investigation of Compound 5

The three‐dimensional electronic surfaces of compound 5 (Figure 8) provide a qualitative picture of the interaction features that may accompany its antibacterial activity. On the MEP map, negative potential is concentrated around carbonyl/phenoxy oxygen atoms and other heteroatom‐rich regions, whereas positive potential is associated mainly with polarized NH/OH hydrogens; together, these regions define complementary hydrogen‐bond acceptor and donor sites [53]. The resulting surface is both polar and aromatic, which may assist recognition in a protein pocket and interaction with a bacterial membrane. NCI‐RDG visualization indicates several weak‐contact regions rather than one dominant interaction, and the ELF/LOL maps show localized electron‐rich centers alongside delocalized π density. This combination is consistent with multiple donor/acceptor positions and possible π‐associated contacts. Thus, the activity of 5 can reasonably be related to the coexistence of accessible hydrogen‐bonding functions, delocalized aromatic surfaces, and an amphiphilic charge distribution; differences in envelope permeability and target accessibility may contribute to the weaker responses observed for *Enterococcus faecalis* and *Pseudomonas aeruginosa* [66].

**FIGURE 8:**
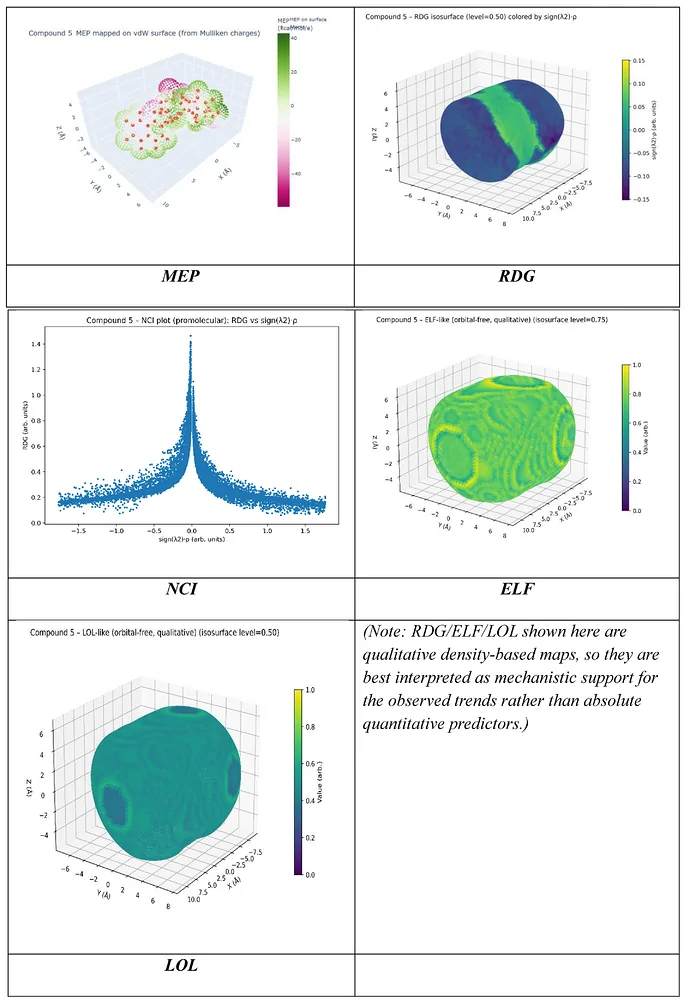
Three‐dimensional electronic‐surface analyses of compound 5: molecular electrostatic potential (MEP) mapped onto the van der Waals surface; NCI‐RDG isosurface (*s* = 0.50) colored according to sign(λ_2_)ρ; NCI scatter plot of RDG versus sign(λ_2_)ρ; electron localization function (ELF) surface; and localized orbital locator (LOL) surface.

## 6 Conclusion

The study therefore integrates activation‐dependent green synthesis, mechanistic interpretation, and antibacterial lead identification, with compound 5 emerging as the principal scaffold for subsequent optimization. The present results show that the physical activation mode can determine which annulation pathway is followed by a common fused‐heterocycle precursor. With reaction time, temperature, and reagent composition held constant, US, MW, and combined MW/US treatment selectively produced 5, 6, and 7, respectively. The three modes therefore acted as more than rate‐acceleration tools; they altered the relative preference for cyclization, tautomerization, dehydration, and further annulation. This activation‐dependent product divergence constitutes the principal synthetic and mechanistic contribution of the study.

Biologically, compound 5 was the strongest member of the synthesized series. Its nitrogen‐rich fused core combines accessible polar functions with hydrophobic aromatic surfaces, and its favorable MIC profile was supported by the docking and MD analyses. The DFT/Mulliken results also provide a chemically consistent picture of the reactive centers involved in the synthetic ring‐closure steps. These observations make 5 a reasonable starting scaffold for subsequent antibacterial optimization, although the current evidence remains an initial in vitro assessment and gram‐scale synthetic validation has not yet been demonstrated. Overall, the work establishes a link between activation‐controlled greener synthesis, mechanistic interpretation, and early antibacterial lead selection.

## Author Contributions

The authors jointly contributed to study conception and design. The authors undertook experimental preparation, data acquisition, and analysis, and the Deputyship for Research and Innovation, Ministry of Education, Saudi Arabia, provided financial support for this study; every author reviewed and approved the final version of the manuscript.

## Conflicts of Interest

The authors declare no conflicts of interest.

## Supporting information




**Supporting File 1**: cbdv71844‐sup‐0001‐SuppMat.docx

## Data Availability

The data that supports the findings of this study are available from the corresponding author upon a reasonable request.
