# Supplementary material for: Green MW/US‐Assisted Construction of Pyrano[2,3‐b]pyridines and Pyrazolo[3,4‐d]pyrimidinones: Pathway Control and Antibacterial Evaluation
Source: Chem Biodivers. 2026 Oct 21;23(10):e71844. doi: 10.1002/cbdv.71844 (PMC13685488; doi:10.1002/cbdv.71844)
Supplement: Supplementary file 1 [file CBDV-23-e71844-s001.docx]

***Supplementary Data:***

***Table.S1:All compounds characterization:***

| Compound | Physical state/color | Yield (%) | m.p. (°C) | IR, νmax (cm⁻¹) | 1H NMR, δ ppm | 13C NMR, δ ppm | EI-MS, m/z | Elemental analysis |
| --- | --- | --- | --- | --- | --- | --- | --- | --- |
| 1 | Pale-yellow crystals | 99 | 88–90 | 1679 C=O, 1600 C=C | 3.59 OCH₃; 6.80–8.02 Ar–H; 9.22 OH | 55.0–188.9 | 304 [M]⁺ | Calcd/Found for C₂₀H₁₆O₃ |
| 2a | Yellow crystals | 85 | 176–178 | 3479 OH, 1732/1669 C=O | 1.21 CH₃; 4.14 CH₂; 8.71 NH₂; 9.41 OH | 29.0–167.0 | 414 [M]⁺, 388 [M−26]⁺ | Calcd/Found for C₂₅H₂₂N₂O₄ |
| 2b | Light-yellow crystals | 84 | 184–186 | 3316 OH, 1733/1725/1674 C=O | 1.03 CH₃; 2.28 CH₃; 3.67 OCH₃; 8.95–8.97 OH | - | 413 [M]⁺ | Calcd/Found for C₂₆H₂₃NO₄ |
| 2c | Light-yellow crystals | 71 | 176–178 | 3411 OH, 1733 C=O | 0.95 CH₃; 3.58 OCH₃; 10.02 OH | 31.1–166.7 | 415 [M]⁺ | Calcd/Found for C₂₅H₂₁NO₅ |
| 3a | Light-yellow crystals | 82 | 176–178 | 3404/3324 NH₂, 2270 CN, 1734 C=O | 7.48–7.86 Ar–H; 8.14 NH₂; 9.26 OH | 61.4–168.7 | 435 [M]⁺ | Calcd/Found for C₂₆H₁₇N₃O₄ |
| 3b | Yellow crystals | 90 | 184–186 | 3211 OH, 1720 C=O | 2.21 CH₃; 2.53 COCH₃; 4.01 OCH₃; 9.32 OH | 42.1–202.3 | 451 [M]⁺ | Calcd/Found for C₂₈H₂₁NO₅ |
| 3c | Light-yellow crystals | 84 | 184–186 | 3428 OH, 1736/1676 C=O | 0.95 CH₃; 3.58 OCH₃; 5.92 CH; 9.25 OH | - | 483 [M]⁺, 453 [M−30]⁺ | Calcd/Found for C₂₈H₂₁NO₇ |
| 4a | Light-yellow crystals | 77 | 176–178 | 3294/3183 NH/NH₂, 1672–1663 C=O, 1613 C=N | 3.38 OCH₃; 8.14 NH₂; 9.41 OH; 10.86 NH | 59.4–166.7 | 467 [M]⁺, 388 [M−79]⁺ | Calcd/Found for C₂₆H₂₁N₅O₄ |
| 4b | Light-yellow crystals | 82 | 184–186 | 3045 Ar–H, 1674 C=O | 2.51 CH₃; 3.35 OCH₃; 9.41 OH; 11.21 NH | Not reported | 465 [M]⁺ | Calcd/Found for C₂₈H₂₃N₃O₄ |
| 5 | Light-yellow crystals | 88 | 176–178 | 3404 OH, 3310 NH, 1686–1663 C=O, 1612 C=N | 1.48 CH₃; 3.28 OCH₃; 4.91 pyridine-H; NH/OH signals | 20.63–166.88 | 491 [M]⁺, 424 [M−67]⁺ | Calcd/Found for C₂₈H₂₁N₅O₄ |
| 6 | Light-yellow crystals | 85 | 176–178 | 3434 OH, 3330/3164/3113 NH, 1673/1654 C=O | 2.07 CH₃; 3.18 OCH₃; 6.19 pyridine-H; 11.47 NH | 20.63–174.11 | 491 [M]⁺, 428 [M−63]⁺ | Calcd/Found for C₂₈H₂₁N₅O₄ |
| 7 | Yellow crystals | 85 | 176–178 | 3468/3345 NH/OH, 1657 C=O, 1631–1619 C=N | 2.40 CH₃; 3.37 OCH₃; 4.51 oxazine-H; NH/OH signals | 10.44–191.62 | 491 [M]⁺, 487 [M−4]⁺ | Calcd/Found for C₂₈H₂₁N₅O₄ |

The EI-MS data provided additional evidence for the proposed molecular structures. The molecular-ion peaks were observed for all synthesized compounds and agreed with the calculated molecular weights. In addition, selected compounds showed characteristic fragment ions arising from loss of small neutral fragments or cleavage around the ester, carbonyl, pyranone, pyrazolone, oxazine, and pyrazolo-pyrimidinone units. These fragmentation pathways support the successful formation of the proposed fused heterocyclic frameworks.

Table.S2:mass fragmentation of all synthezied compounds

| Compound | Molecular formula | MW | Reported EI-MS peaks, m/z | Proposed Suggested fragmentation pathway |
| --- | --- | --- | --- | --- |
| 1 | C₂₀H₁₆O₃ | 304.34 | 304 [M]⁺ | Stable chalcone molecular ion; fragmentation may occur through cleavage of the α,β-unsaturated carbonyl system and formation of methoxy-naphthyl/phenolic aromatic ions. |
| 2a | C₂₅H₂₂N₂O₄ | 414.46 | 414 [M]⁺, 388 [M − 26]⁺ | Loss of a small neutral fragment of 26 mass units, possibly C₂H₂/HCN-type rearranged fragment, followed by stabilization of the nicotinate aromatic cation. |
| 2b | C₂₆H₂₃NO₄ | 413.47 | 413 [M]⁺ | Stable nicotinate molecular ion; expected fragmentation through ester-side-chain cleavage, loss of ethoxy/methyl fragments, and aromatic cation stabilization. |
| 2c | C₂₅H₂₁NO₅ | 415.45 | 415 [M]⁺ | Stable pyridone-carboxylate molecular ion; expected cleavage around ester carbonyl and pyridone ring with formation of conjugated aromatic fragments. |
| 3a | C₂₆H₁₇N₃O₄ | 435.44 | 435 [M]⁺ | Confirms pyrano[2,3-b]pyridine carbonitrile framework; fragmentation may involve cleavage near the CN-bearing pyranone ring and loss of small N-containing fragments. |
| 3b | C₂₈H₂₁NO₅ | 451.48 | 451 [M]⁺ | Stable acetylated pyrano[2,3-b]pyridine ion; likely fragmentation through acetyl group cleavage and rupture of the pyranone carbonyl region. |
| 3c | C₂₈H₂₁NO₇ | 483.48 | 483 [M]⁺, 453 [M − 30]⁺ | fragment ion  Loss of 30 mass units, plausibly CH₂O or a small oxygenated fragment, supporting the presence of methoxy/ester-containing pyranopyridine structure. |
| 4a | C₂₆H₂₁N₅O₄ | 467.49 | 467 [M]⁺, 388 [M − 79]⁺ | major fragment ion  Loss of a 79-mass-unit neutral fragment from the hydrazine-derived heterocyclic framework; supports formation of the N-rich pyrazolone/pyridone system. |
| 4b | C₂₈H₂₃N₃O₄ | 465.51 | 465 [M]⁺ | Confirms the methyl-pyrazolylidene pyridone structure; expected fragmentation through cleavage of the pyrazolone side chain and aromatic stabilization. |
| 5 | C₂₈H₂₁N₅O₄ | 491.51 | 491 [M]⁺, 424 [M − 67]⁺ | major fragment ion  Loss of a 67-mass-unit neutral fragment, consistent with cleavage within the pyrazolo[3,4-d]pyrimidinone annulated system; supports formation of compound 5. |
| 6 | C₂₈H₂₁N₅O₄ | 491.51 | 491 [M]⁺, 428 [M − 63]⁺ | major fragment ion  Loss of a 63-mass-unit fragment from the oxazinyl/iminopyrazolidinone system, supporting the formation of the pyrido[3,2-e][1,3]oxazine derivative. |

Figure S1: Outline ^1^H-NMR of compound 1

Figure S2: Outline ^1^H-NMR of compound 2a

Figure S3: Outline ^1^H-NMR of compound 2b

Figure S4: Outline ^1^H-NMR of compound 2c

Figure S5: Outline ^1^H-NMR of compound 3a

Figure S6: Outline ^1^H-NMR of compound 3b

Figure S7: Outline ^1^H-NMR of compound 3c

Figure S8: Outline ^1^H-NMR of compound 4a

Figure S9: Outline ^1^H-NMR of compound 4b

Figure S10: Outline ^1^H-NMR of compound 5

Figure S11: Outline ^1^H-NMR of compound 6

Figure S12: Outline ^1^H-NMR of compound 7

Figure S13: Outline MS of compound 2a

Figure S14: Outline IR of compound 3a

Figure S15: Outline IR of compound 6

Figure S16: Outline MS of compound 7

Figure S17: Outline IR of compound 3a

Figure S18: Outline MS of compound 2a

Figure S19: Outline IR of compound 4a
